# Supplementary material for: Health-related educational inequalities in paid employment across 26 European countries in 2005–2014: repeated cross-sectional study
Source: BMJ Open. 2019 Jun 1;9(5):e024823. doi: 10.1136/bmjopen-2018-024823 (PMC6549613; doi:10.1136/bmjopen-2018-024823)
Supplement: Supplementary data [file bmjopen-2018-024823supp001.pdf]

Supplementary table 1 | Absolute differences in paid employment between persons with and without a chronic illness by educational level in European regions between 2005-2014 - male

Region in Europe	Absolute difference (%)									
	2005	2006	2007	2008	2009	2010	2011	2012	2013	2014
**Low education**										
Nordic	26.1	27.0	25.2	25.7	26.7	32.9	29.6	32.6	31.4	32.9
Continental	22.9	23.0	24.5	25.9	24.3	24.6	29.2	24.7	25.2	26.1
Anglo-Saxon	38.7	35.6	37.2	40.4	27.3	32.2	38.9	34.6	34.7	35.3
Southern	23.7	24.5	25.6	26.1	23.5	22.1	22.1	21.7	19.6	21.5
Eastern	28.6	31.4	36.9	35.8	30.9	32.5	32.7	35.5	32.8	33.5
**Intermediate education**										
Nordic	19.2	18.5	18.4	16.8	18.8	20.8	19.0	18.9	19.1	18.6
Continental	16.4	15.9	15.5	16.4	18.2	17.2	16.6	17.3	17.0	17.2
Anglo-Saxon	17.9	17.7	18.5	18.5	18.0	23.9	20.3	23.7	25.7	17.1
Southern	12.4	12.4	12.7	13.3	14.9	13.7	11.9	11.2	9.6	11.2
Eastern	26.0	26.6	27.5	28.7	27.1	27.8	26.9	27.5	25.3	25.5
**High education**										
Nordic	10.7	8.2	8.6	8.0	9.3	7.3	6.6	9.3	10.7	11.1
Continental	11.4	9.5	8.8	8.3	7.7	8.3	7.8	7.7	8.7	8.2
Anglo-Saxon	12.1	11.5	10.6	14.6	14.4	16.4	11.9	15.8	13.4	13.4
Southern	9.3	10.5	6.6	8.9	10.9	7.8	6.1	6.9	5.5	5.0
Eastern	10.5	11.9	9.8	12.3	11.2	12.7	10.2	12.1	11.6	9.8

Supplementary table 2 | Absolute differences in paid employment between persons with and without a chronic illness by educational level in European regions between 2005-2014 - female

Region in Europe	Absolute difference (%)									
	2005	2006	2007	2008	2009	2010	2011	2012	2013	2014
**Low education**										
Nordic	31.6	34.1	33.3	31.0	32.9	34.0	34.9	42.0	41.1	31.0
Continental	15.9	15.3	13.6	14.7	16.3	19.0	18.4	19.0	21.9	20.4
Anglo-Saxon	25.7	20.5	27.9	25.6	23.3	23.6	25.7	29.3	28.9	29.8
Southern	8.6	10.1	10.7	11.1	9.1	9.6	10.8	8.0	7.7	10.0
Eastern	20.2	20.7	21.9	19.4	22.1	20.5	19.8	24.6	23.4	23.7
**Intermediate education**										
Nordic	18.9	18.2	21.8	20.7	18.9	17.4	19.4	20.3	16.7	20.1
Continental	10.7	11.3	12.0	12.7	13.3	13.9	12.7	14.0	13.7	15.2
Anglo-Saxon	14.5	13.9	14.2	18.0	17.4	14.7	13.0	18.9	21.8	22.5
Southern	5.2	4.8	9.1	5.7	5.5	6.8	5.2	5.6	8.8	6.5
Eastern	19.1	19.9	21.8	21.8	22.0	22.1	21.8	22.9	21.1	20.6
**High education**										
Nordic	8.1	11.8	11.4	9.1	9.6	9.0	6.9	6.2	9.2	9.6
Continental	8.2	8.7	7.9	9.0	6.2	7.4	8.8	7.2	11.7	9.4
Anglo-Saxon	13.7	11.5	13.4	11.9	9.3	9.0	8.1	13.0	14.4	15.8
Southern	4.5	9.4	6.2	2.5	2.8	3.1	3.6	2.6	3.5	5.8
Eastern	10.0	8.2	9.9	8.6	8.0	9.2	9.1	8.4	7.5	6.4

Supplementary table 3 | Relative differences in paid employment between persons with and without a chronic illness by educational level in European regions between 2005-2014 - male

Region in Europe	Relative difference (PR)									
	2005	2006	2007	2008	2009	2010	2011	2012	2013	2014
**Low education**										
Nordic	1.43	1.43	1.39	1.39	1.45	1.62	1.53	1.60	1.58	1.61
Continental	1.40	1.40	1.43	1.46	1.43	1.45	1.56	1.45	1.46	1.49
Anglo-Saxon	1.86	1.74	1.83	2.05	1.66	1.88	2.14	1.95	2.02	1.79
Southern	1.40	1.42	1.43	1.44	1.41	1.38	1.40	1.41	1.38	1.43
Eastern	1.80	1.89	2.05	1.93	1.85	1.96	1.99	2.11	1.95	1.94
**Intermediate education**										
Nordic	1.26	1.25	1.24	1.22	1.26	1.29	1.26	1.26	1.26	1.25
Continental	1.23	1.22	1.21	1.22	1.25	1.24	1.23	1.24	1.23	1.24
Anglo-Saxon	1.24	1.23	1.25	1.25	1.26	1.39	1.31	1.37	1.42	1.25
Southern	1.16	1.16	1.16	1.17	1.20	1.18	1.16	1.15	1.13	1.16
Eastern	1.46	1.46	1.46	1.48	1.47	1.50	1.47	1.48	1.42	1.42
**High education**										
Nordic	1.13	1.09	1.10	1.09	1.11	1.08	1.07	1.11	1.13	1.13
Continental	1.14	1.11	1.10	1.09	1.09	1.10	1.09	1.09	1.10	1.09
Anglo-Saxon	1.15	1.14	1.13	1.18	1.19	1.22	1.15	1.21	1.17	1.17
Southern	1.11	1.13	1.08	1.11	1.13	1.09	1.07	1.08	1.07	1.06
Eastern	1.12	1.14	1.11	1.15	1.13	1.16	1.12	1.15	1.14	1.11

Supplementary table 4 | Relative differences in paid employment between persons with and without a chronic illness by educational level in European regions between 2005-2014 - female

Region in Europe	Relative difference (PR)									
	2005	2006	2007	2008	2009	2010	2011	2012	2013	2014
**Low education**										
Nordic	1.63	1.67	1.64	1.58	1.66	1.72	1.74	2.02	1.96	1.65
Continental	1.44	1.38	1.32	1.33	1.37	1.45	1.44	1.45	1.53	1.48
Anglo-Saxon	1.84	1.59	2.02	1.86	1.79	1.86	2.04	2.16	2.22	1.89
Southern	1.27	1.31	1.32	1.31	1.25	1.26	1.29	1.21	1.21	1.28
Eastern	1.70	1.77	1.78	1.63	1.77	1.71	1.68	1.94	1.80	1.76
**Intermediate education**										
Nordic	1.28	1.27	1.32	1.30	1.27	1.25	1.28	1.30	1.24	1.30
Continental	1.19	1.18	1.20	1.21	1.21	1.22	1.19	1.22	1.21	1.24
Anglo-Saxon	1.25	1.23	1.23	1.30	1.30	1.26	1.22	1.34	1.41	1.44
Southern	1.08	1.08	1.16	1.09	1.09	1.11	1.09	1.10	1.16	1.11
Eastern	1.38	1.39	1.42	1.41	1.42	1.43	1.41	1.44	1.38	1.37
**High education**										
Nordic	1.10	1.15	1.14	1.11	1.12	1.11	1.08	1.07	1.11	1.12
Continental	1.11	1.12	1.10	1.12	1.08	1.09	1.11	1.09	1.15	1.12
Anglo-Saxon	1.20	1.16	1.19	1.16	1.13	1.13	1.11	1.19	1.21	1.23
Southern	1.05	1.12	1.07	1.02	1.03	1.04	1.04	1.03	1.04	1.08
Eastern	1.11	1.09	1.12	1.10	1.10	1.11	1.11	1.10	1.09	1.08
