# Supplementary material for: Health-related educational inequalities in paid employment across 26 European countries in 2005–2014: repeated cross-sectional study
Source: BMJ Open. 2019 Jun 1;9(5):e024823. doi: 10.1136/bmjopen-2018-024823 (PMC6549613; doi:10.1136/bmjopen-2018-024823)
Supplement: Author's manuscript [file bmjopen-2018-024823.draft_revisions.pdf]

BMJ Open

BMJ Open is committed to open peer review. As part of this commitment we make the peer review history of every article we publish publicly available.

When an article is published we post the peer reviewers' comments and the authors' responses online. We also post the versions of the paper that were used during peer review. These are the versions that the peer review comments apply to.

The versions of the paper that follow are the versions that were submitted during the peer review process. They are not the versions of record or the final published versions. They should not be cited or distributed as the published version of this manuscript.

BMJ Open is an open access journal and the full, final, typeset and author-corrected version of record of the manuscript is available on our site with no access controls, subscription charges or pay-per-view fees (http://bmjopen.bmj.com).

If you have any questions on BMJ Open's open peer review process please email editorial.bmjopen@bmj.com

BMJ Open

Health-related educational inequalities in labour force participation across 26 European countries in 2005-2014: a trend analysis

Journal:	*BMJ Open*
Manuscript ID	bmjopen-2018-024823
Article Type:	Research
Date Submitted by the Author:	18-Jun-2018
Complete List of Authors:	Schram, Jolinda; Erasmus MC afdeling Maatschappelijke GezondheidsZorg, Public Health<br>Schuring, Merel; Erasmus MC<br>Oude Hengel, Karen; Erasmus MC; Netherlands Organization for Applied Scrientific Research TNO<br>Burdorf, Alex; Erasmus MC, Department of Public Health
Keywords:	Trend analysis, Social inequalities, Chronic illness, Employment

SCHOLARONE™
Manuscripts

For peer review only

Title **Health-related educational inequalities in labour force participation across 26 European countries in 2005-2014: a trend analysis**

Authors' names Jolinda LD Schram[1], Merel Schuring[1], Karen M Oude Hengel[1,2], Alex Burdorf[1]

Corresponding author Correspondence to: Merel Schuring, Erasmus University Medical Center, Department of Public Health, P.O. Box 2040, 3000 CA Rotterdam, The Netherlands, m.schuring@erasmusmc.nl , +31107038993

Affiliation [1] Erasmus University Medical Center, Department of Public Health, P.O. Box 2040, 3000 CA Rotterdam, The Netherlands.
[2] Netherlands Organization for Applied Scientific Research TNO, P.O. Box 3005, 2301 DA Leiden, The Netherlands.

Key words Social inequalities, Employment, Chronic illness, Trend analysis

Word count 3,680

For peer review only

ABSTRACT

Objective The study investigates trends in health-related inequalities in labour force participation among men and women in different educational groups in 26 countries in five European regions.

Design The EU Statistics on Income and Living Conditions (EU-SILC) repeated cross-sectional annual data (n=1,844,915) were used to determine trends in labour force participation between 2005–2014 among men and women with and without a chronic illness by educational level. Five European regions were defined based on welfare state regimes.

Primary and secondary outcome measures Absolute differences were expressed by absolute differences in proportion in paid employment between participants with and without a chronic illness, using linear regression. Relative differences were expressed by prevalence ratios in paid employment, using a Cox proportional hazard model. Linear regression was used to examine the trends of inequalities.

Results Participants with a chronic illness had consistently lower labour force participation than those without illnesses. Educational inequalities were substantial with absolute differences larger within lower educated (men 21-35%, women 10-31%) than within higher educated (men 5-13%, women 6-16%). Relative differences showed that low educated men with a chronic illness were 1.4-1.9 times (women 1.3-1.8 times) more likely to be out of paid employment than low educated persons without a chronic illness, whereas this was 1.1-1.2 among high educated men and women. In the Nordic, Anglo-Saxon and Eastern regions these health-related educational inequalities were more pronounced than in the Continental and Southern region. For most regions, absolute health-related educational inequalities in labour force participation were generally constant, whereas relative inequalities increased, especially among low educated persons.

Conclusions Despite policies and programmes, men and women with a chronic illness have considerable less access to the labour market than their healthy colleagues, especially among lower educated persons. This exclusion from the labour market will reduce personal income and, thus, increase health inequalities.

For peer review only

For peer review only - http://bmjopen.bmj.com/site/about/guidelines.xhtml

Strengths and limitations of the study

- This is the first study to present information on trends in health-related educational inequalities in paid employment among persons in different European regions
- The repeated cross-sectional survey presents descriptive evidence that the difference in labour participation between those with a chronic disease and those without a chronic disease widens with lower educational attainment.
- The effects of having a chronic illness on being employed (selection) and unemployment causing ill health such as chronic illness (causation), cannot be distinguished by using cross-sectional data.
- Although the outcomes of EU-SILC are harmonized, comparisons in self-rated health measures between different cultures need to be made with caution.

For peer review only

For peer review only - http://bmjopen.bmj.com/site/about/guidelines.xhtml

Introduction

Life expectancy continues to increase and will ensure that the old-age dependency ratio, the number of elderly people as a share of those of working age, will rise steadily from 29.4 in 2016 to 50.3 by 2050 in Europe (1, 2). In response, many countries have raised the statutory retirement age (3). The challenge is ensuring that those of working age are actually participating in the labour force (4). Health related exclusion from the labour market is present in many countries (5, 6), whereby workers with a chronic illness are at increased risk of becoming disabled or unemployed. Many countries are developing policies to retain persons with a chronic illness in paid employment (7).

Educational inequalities in morbidity, such as chronic health conditions, are well documented across European countries (8). Review studies have shown that the presence of a chronic disease is an important barrier for entering and maintaining paid employment (5). Some studies have suggested that the effect of chronic illness on maintaining paid employment differs by socioeconomic position. Bartley and Owen reported that workers with a chronic disease in lower socioeconomic position had more difficulties staying in paid employment than those with a higher socioeconomic position during times of economic crisis (9). In Sweden and the United Kingdom labour market attachment for those with a chronic illness is reduced , with the effect being substantially stronger in the United Kingdom than in Sweden (10, 11). Burstrom et al. suggested that national labour market policies may modify the differential effects across socioeconomic position of chronic illness on paid employment (12).

The fivefold welfare state regime typology (Nordic, Continental, Anglo-Saxon, Southern and Eastern) has been widely used in public health research in recent years (13, 14). This typology reflects general differences in employment protection and income security. There is no clear agreement across studies whether welfare regimes influence the labour attachment of persons with a chronic illness. Van de Wel et al. showed that non-employment rates for persons with a longstanding illness as well as a low educational level were particularly high in Anglo-Saxon and Eastern welfare state regimes, compared to the Nordic welfare state regime (15). The same authors reported that welfare states with generous unemployment and social insurance benefits had both smaller absolute and relative health inequalities in sickness absence (16). Another European study by Reeves et al. suggested that employment protection policies may partly counteract the poor position of sick or disabled persons on the labour market (17). However, a comparative analysis by Dahl et al., concluded that health inequalities in itself were not consistently smaller in Nordic countries than in other European welfare state regimes (18).

Few studies have documented the trends over time in socioeconomic differences in labour force participation between persons with and without a chronic illness. A repeated cross sectional study over 25 years in Britain showed that within the lower occupational class persons with a limiting illness had greater and more rapidly widening gaps in labour force participation than in the higher occupational class (19). A comparison between the Nordic countries and the United Kingdom showed that, since the mid-1990s, there has been a deterioration in paid employment among persons with limiting long standing illness with a low education, even when the economy was doing well (20).

There is limited information on trends in health-related educational inequalities in employment among persons in various welfare state regimes, since most studies have focused on a single country or compared a selected group of countries. Therefore, the aims of this paper are to examine absolute

For peer review only - http://bmjopen.bmj.com/site/about/guidelines.xhtml

and relative differences in labour force participation between persons with and without a chronic illness within each educational group in five European regions and to describe trends in socioeconomic differences in labour force participation between people with and without a chronic illness within European countries.

For peer review only

For peer review only - http://bmjopen.bmj.com/site/about/guidelines.xhtml

Methods

Data

A repeated cross-sectional study was conducted with the EU-SILC survey (European Union Statistics on Income and Living Conditions). The EU-SILC is a survey questionnaire designed to provide comparable information across the EU and cross-sectional data from 2005 to 2014 was used from 26 countries that participated in EU-SILC since 2005 (Denmark, Finland, Iceland, Norway, Sweden, Austria, Belgium, Germany, France, Luxembourg, The Netherlands, Cyprus, Greece, Italy, Portugal, Spain, Ireland, United Kingdom, Czech Republic, Estonia, Hungary, Latvia, Lithuania, Poland, Slovakia and Slovenia). Detailed information on the EU-SILC study design can be found elsewhere (21).

Respondents were selected with information on work status, chronic illness, educational background, age, and gender. Our study comprises 179,724 individuals aged 30–59 years in 2014. For the trend analysis 1,844,915 individuals were selected aged 30-59 years between 2005-2014. The age limit of 59 years was applied because a number of countries have an official retirement age of 60, thereby greatly reducing the proportion of employed persons after 60 years. The age limit of 30 years was used to exclude potential confounding due to different educational pathways between those with a chronic illness and those without.

Labour force participation

Labour force participation was classified into six mutually exclusive categories: Employed (working full or part-time, self-employed full or part-time), unemployed, economically inactive (fulfilling domestic tasks and care responsibilities, and other inactivity), retired, disabled and other (student, in compulsory military community or service). For the main analysis a dichotomized variable was used: paid employment or not (all other options).

Chronic illness

The question *Do you have any longstanding illness or health problem?* was used to distinguish between people with or without a chronic illness.

Sociodemographic variables

Three sociodemographic variables were used in the study: age, gender, and education. Age was categorized into three 10-year age groups: 30-39, 40-49 and 50-59 years. Educational level was recorded as the highest level of education completed by the respondent. We coded education according to the 1997 International Standard Classification of Education (ISCED-97) and categorized as low (0-2: pre-primary, primary and lower secondary education), intermediate (3-4: upper secondary education/post-secondary non tertiary) and high (5-6: tertiary education).

Regions

The European countries were categorized into five different regions, based on relative comparability of welfare state regimes and geography (13). The Nordic region included Denmark, Finland, Iceland, Norway, and Sweden. The Continental region included Austria, Belgium, Germany, France, Luxembourg, and The Netherlands. The Southern region included Cyprus, Greece, Italy, Portugal, and Spain. The Anglo-Saxon countries included Ireland and the United Kingdom. The Eastern region included the Czech Republic, Estonia, Hungary, Latvia, Lithuania, Poland, Slovakia, and Slovenia.

For peer review only - http://bmjopen.bmj.com/site/about/guidelines.xhtml

Statistical analysis

The statistical analysis focuses on both absolute and relative differences in labour force participation among those with and without a chronic disease across different educational groups. The absolute difference depicts the reduction of being in paid employment among persons with a chronic illness and is of special interest as it presents the impact on the total population in %point employment rate. The relative difference presents the prevalence ratio as a measure of the strength of the association between having a chronic illness and being out of paid employment. Absolute and relative inequalities may differ because levels of labour force participation are generally higher in higher educated groups.

Annual sample weights were used to estimate the prevalence of labour market participation that are representative for the countries included in this study. For each region the association of having a chronic illness with labour force participation within each educational group was analysed using two different models. A linear regression model was used to calculate absolute differences in labour force participation between persons with and without a chronic illness. Cox regression analysis with a robust estimate was applied to calculate relative differences. Cox regression analysis was applied instead of logistic regression, since the odds ratio will overestimate the prevalence ratio due to the high prevalence of being in paid employment (22).

The linear regression model to calculate absolute difference can be written as:

$$y = \alpha + \beta_1 low + \beta_2 intermediate + \beta_3 low\_chronic + \beta_4 intermediate\_chronic + \beta_5 high\_chronic + \beta_6 age$$

where *y* is a dichotomous variable for being employed; *α* is an intercept, two dummy variables to compare low and intermediate education with high education, and three additional dummy variables to compare those with a chronic illness to those without in each education group. Adjustment for age was done by default.

The Cox proportional hazard model to calculate relative differences can be written as:

$$h(t,x) = h_0(t)\exp\left(\sum_{k=1}^{p} \beta_1 low + \beta_2 intermediate + \beta_3 low\_chronic + \beta_4 intermediate\_chronic + \beta_5 high\_chronic + \beta_6 age\right)$$

where *h(t,…)* is the likelihood at time *t* that a person is not employed, two dummy variables for low and intermediate education to compare to high education, and three dummy variables to compare those with a chronic illness in each education group. Adjustment for age was done by default.

The time period for all persons was set at a constant level (value 1) to estimate the prevalence ratio (PR) (22, 23). In all analyses, variables were recoded so a PR above 1 indicates an increased likelihood of being out of paid employment with corresponding 95% confidence intervals (95% CI). The associations were adjusted for age and stratified by region and sex. Significance was determined by $P < 0.05$.

Trend analysis in the five regions

For determining the trends in absolute and relative health-related inequalities in labour force participation over 10 years in each region, we used linear regression analyses with the absolute gap

For peer review only - http://bmjopen.bmj.com/site/about/guidelines.xhtml

and prevalence ratios in each educational group as dependent variable in each survey year. The analysis was stratified by region and gender. The year of data collection was used as the independent variable.

Health-related educational inequalities

In order to estimate educational inequalities in the relative differences in labour force participation among persons with a chronic illness a counterfactual scenario was introduced. In this scenario it is assumed that the relative difference in labour force participation between those with a chronic illness and those without in the lower educational group can be reduced to the relative difference observed in the higher educational group (24). The estimation of the excess relative difference in paid employment for chronic illness status in lower educated workers is a fair reflection of educational inequalities between low and high educated persons. The Cox proportional hazard model was used to estimate this excess relative difference by the formula: (bèta for relative difference in the low educated group – bèta for relative difference in the high educated group)/(bèta of the relative difference in the low educated group).

The main analysis was performed using Stata statistical software version 14 (Stata-Corp, College Station, Texas). The estimates of paid employment per country and the trend analysis were conducted in IBM SPSS statistical software version 22 (SPSS Inc, Chicago, IL, USA).

Patient involvement

Not applicable, we have used available datasets from Eurostat for secondary data analysis.

For peer review only - http://bmjopen.bmj.com/site/about/guidelines.xhtml

Results

The study population consisted of 179,724 participants of whom half were women, the largest group was intermediately educated, 75% were employed, and 27% had a chronic illness. The Eastern region had a large intermediate educated group (64%), whereas the Southern and Anglo-Saxon regions had a relatively large low educated group (28 and 38%, respectively) (Table 1). The Southern region had the lowest labour force participation, and the lowest percentage of people with a chronic illness. The Nordic region had the highest employment, but also the highest prevalence of people with a chronic illness.

Labour force participation over the period 2005-2014 among men without a chronic illness remained stable for intermediate and high educated persons, but decreased by about 5% among those with lower education (Figure 1A). Within each educational group, labour force participation among men with a chronic illness was substantially lower than among men without illness, with the largest difference among the lowest educated persons. For women the labour force participation increased slightly for most groups over the period 2005-2014 (Figure 1B). Women had a consistently lower labour force participation than men with increasing differences by lower educational level. Women with a chronic disease had substantially lower labour force participation than women without a chronic illness.

The absolute differences in labour force participation between persons with and without a chronic illness increased with lower educational level, among men and women (Table 2). The absolute gaps for men were larger than for women. For low educated persons the absolute differences in labour force participation between men with and without a chronic illness were higher in the Nordic, Anglo-Saxon and Eastern region (32.9-35.3%) than in the Continental and Southern region (21.5-26.1%). Among women a comparable pattern was observed.

A similar pattern was found for relative differences in health-related labour force participation (Table 3). Overall, low educated men with a chronic illness were 1.4 to 1.9 times more likely to be out of paid employment than low educated men without a chronic illness, whereas this was 1.1 to 1.2 times within the high educational group. Relative differences in labour force participation among low educated men without and with a chronic illness were the highest in the Anglo-Saxon and Eastern region (1.79-1.94). For men with high education modest regional differences were found in labour force participation between those without and with a chronic illness. Comparable patterns were observed for women.

Trends in absolute differences in labour force participation showed stable inequalities for men in all educational groups in most regions (Table 4). Among men, significant narrowing of absolute health-related inequalities was observed for all educational categories in the Southern region over the period 2005-2014. For women, significant widening of absolute health-related inequalities were observed for low and intermediate educated women in the Continental and Anglo-Saxon region. In the Eastern region significant widening in inequalities was observed for low educated women, while narrowing inequalities were observed for high educated women.

Trends in relative differences in labour force participation were constant for high educated men and widened for low educated men (Table 5). Among men, significant widening of relative differences were observed for lower educated persons in the Nordic and Continental region of respectively 1% and 2.6% over the period 2005-2014. Significant narrowing of relative health-related inequalities was observed for higher educated men in the Continental and Southern region (-0.3% and -0.6%). Trends for women were similar. In most regions relative health-related inequalities were widening for low educated women (between 1.0% and 3.7%), although in the Southern region there was a small narrowing (-0.7%). For high educated women there was no clear trend.

For peer review only - http://bmjopen.bmj.com/site/about/guidelines.xhtml

Figure 2 presents the inequalities between low and high educated persons for the influence of a chronic illness on having paid employment within each educational group. The excess relative difference among low educated persons across 26 European countries varied between 50% to 100% among men and between 58% and 100% among women when compared to high educated persons. The variation in educational inequalities in labour market position of those with a chronic disease was larger within European regions than between European regions, indicating substantial differences between countries with supposedly similar welfare regimes.

For peer review only

For peer review only - http://bmjopen.bmj.com/site/about/guidelines.xhtml

Discussion

Within Europe, persons with a chronic disease were less often in paid employment than persons without a chronic disease in the period 2005-2014. The low education group had the largest absolute and relative differences in employment between persons with and without a chronic illness. For men these differences were more pronounced than for women. The Nordic, Anglo-Saxon and Eastern regions had substantially higher relative and absolute differences in labour force participation than in the Continental and Southern region. The relative position on the labour market for persons with a chronic illness worsened in the past 10 years among low educated persons, and remained fairly stable for high educated persons.

The risk of being out of paid employment with a chronic illness is higher for persons with a low education than those with a high education. Previous studies have shown comparable results in specific countries (9, 11, 17, 19) or across Europe in one particular year. (16, 25) Our study expands this knowledge by demonstrating that these patterns are consistent over time and European countries, and by illustrating that health-related educational inequalities in labour force participation have widened rather than narrowed in the past decade.

Men and women with a chronic illness have less access to the labour market than their healthy colleagues, especially among lower educated persons. Possible explanations for the observed educational inequality relate to severity of chronic illnesses and quality of jobs. In this study chronic illness could not be differentiated by diagnosis and severity. It may be hypothesised that lower educated workers more often suffer from chronic diseases with functional limitations than high educated workers (26, 27). Having a chronic illness and functional limitations follows a social gradient in our data (high educated 49%, low educated 73%). The education inequality may also be explained by differences in strenuous work and other job characteristics that may interfere with health problems (28, 29).

A few studies have demonstrated that strenuous work conditions hamper workers with a chronic illness to remain in paid employment. Among Finnish workers physical work load strongly mediated the association between musculoskeletal disorders and exit from paid employment (30). A Dutch study attributed 12% of educational differences in disability benefits to work characteristics (31). Another Dutch study showed that high autonomy, high social support and low psychosocial job strongly moderated the association between chronic health conditions and exit through disability benefits (32). Lower socio-economic jobs more often have unfavourable work characteristics (33). Therefore, the ability to remain in paid employment is reduced for low educated persons with a chronic illness.

Health-related absolute and relative differences in labour force participation across educational groups were consistently lower in the Southern region than in other European regions. A few studies (12, 16, 19) have noted larger absolute and relative differences in labour force participation between persons with and without a chronic illness in regions with low employment protection compared to regions with high employment protection. Our results in the Southern region, which is characterised by high employment protection, corroborates these previous studies. However, the educational inequalities depicted in figure 2 showed a larger variation between countries in the same region than between different regions. This suggests that other social, legal and economic conditions, such as availability of social benefits, also play a role in being in paid employment of people with a chronic illness. More studies are necessary to evaluate the influence of various national indicators of social, cultural and economic conditions on the variation in health-related educational inequalities in labour force participation.

For peer review only - http://bmjopen.bmj.com/site/about/guidelines.xhtml

The absolute and relative disadvantages in labour force participation among those with a chronic disease were more pronounced for men than for women in most welfare states. In the Southern welfare state regime the absolute and relative differences were smaller for women, but in the Nordic welfare state regime the two groups showed similar patterns. These regional differences between gender may partly be explained by the full-employment policies of the Nordic welfare state regime, that facilitate the integration of women into the labour force by providing family supportive services such as child care (34). The high labour force participation of women in Nordic welfare states can contribute to higher relative differences between those with and without a chronic illness compared to the Southern region. This is in line with previous studies stating that in terms of socioeconomic inequalities the relative inequalities do not appear to be smallest in more redistributive countries such as the Scandinavian welfare state, but that the absolute health of all social classes is often better (35).

The trends observed in this study showed that over the period 2005 to 2014 educational inequalities in labour force participation of persons with a chronic illness have widened among low educated persons. In the recent decades precarious employment, such as temporary contracts and part-time work, have become more common in Europe (36, 37). The increase in precarious employment may be partly responsible for increasing educational inequalities in labour force participation, but this particular path of inquiry needs to be studied further.

The trend analysis might be influenced by the distribution of the non-employed and employed groups over time. The Continental and Eastern region have had an increase in labour force participation over the period 2005-2014, while the Southern region had a decrease of employment due to the economic crisis. In periods of high and rapid increase in unemployment rates, workers in good health will also lose their jobs and, thus, the particular influence of ill health on paid employment will attenuate.(38) This mechanism may explain why we found a decreasing trend in the Southern region.

Strengths and limitations

Data were derived from the European Union Statistics on Income and Living Conditions (EU-SILC). A strength of the current study is the use of cross sectional data from a large number of European countries for which the outcomes are harmonized. The repeated cross-sectional surveys present a snapshot of the labour force situation of each region for the respective years, but causal relations cannot be investigated in depth, as other studies with cohort information have done (39, 40). By using cross sectional data in this study the effects of having a chronic illness on being employed (selection) and unemployment causing ill health such as chronic illness (causation), cannot be distinguished.

A limitation of using EU-SILC data is the variation in mode of data collection, translations, and cultural interpretation (16). Further limitations are the use of self-reported health and labour market measures which could vary by country, socioeconomic position and cultural beliefs (13). However, a number of studies have shown that self-rated measurements of health are strongly predictive for objective health measures, such as mortality (41). Comparisons in self-rated health measures between different cultures do need to be made with caution (42).

Policy implications

Men and women with a chronic illness have considerable less access to the labour market than their healthy colleagues, especially among lower educated persons. Despite policies and programmes to improve their position, persons with a chronic illness are still at substantial risk to be excluded from paid employment. Their position in the labour market seems to have worsened rather than improved in the past decade. Persons with a chronic illness without a paid job are at risk for further deterioration of their health and economic marginalisation. More effective policy measures are

For peer review only - http://bmjopen.bmj.com/site/about/guidelines.xhtml

needed to reduce the gap in employment between those with and without a chronic illness, especially in the low education group, in all European regions.

For peer review only

For peer review only - http://bmjopen.bmj.com/site/about/guidelines.xhtml

References

1. Eurostat. Projected old-age dependency ratio: Eurostat; 2013 [updated 11 September 2017. Available from: http://ec.europa.eu/eurostat/tgm/table.do?tab=table&init=1&language=en&pcode=tsdde511&plugin=1.
2. Eurostat. Old-age-dependency ratio: Eurostat; 2017 [updated 11 September 2017. Available from: http://ec.europa.eu/eurostat/tgm/table.do?tab=table&init=1&language=en&pcode=tsdde510&plugin=1.
3. Christensen K, Doblhammer G, Rau R, Vaupel JW. Ageing populations: the challenges ahead. Lancet. 2009;374(9696):1196-208.
4. Spijker J, MacInnes J. Population ageing: the timebomb that isn't? BMJ. 2013;347:f6598.
5. van Rijn RM, Robroek SJ, Brouwer S, Burdorf A. Influence of poor health on exit from paid employment: a systematic review. Occup Environ Med. 2014;71(4):295-301.
6. Carr E, Fleischmann M, Goldberg M, Kuh D, Murray ET, Stafford M, et al. Occupational and educational inequalities in exit from employment at older ages: evidence from seven prospective cohorts. Occup Environ Med. 2018.
7. OECD/EU. Health at a Glance: Europe 2016 - State of Health in the EU Cycle. Paris: 2016.
8. Dalstra JAA, Kunst AE, Borrell C, Breeze E, Cambois E, Costa G, et al. Socioeconomic differences in the prevalence of common chronic diseases: an overview of eight European countries. International Journal of Epidemiology. 2005;34(2):316-26.
9. Bartley M, Owen C. Relation between socioeconomic status, employment, and health during economic change, 1973-93. Brit Med J. 1996;313(7055):445-9.
10. Lindholm C, Burstrom B, Diderichsen F. Class differences in the social consequences of illness? J Epidemiol Commun H. 2002;56(3):188-92.
11. Burstrom B, Holland P, Diderichsen F, Whitehead M. Winners and losers in flexible labor markets: The fate of women with chronic illness in contrasting policy environments - Sweden and Britain. International Journal of Health Services. 2003;33(2):199-217.
12. Burstrom B, Whitehead M, Clayton S, Fritzell S, Vannoni F, Costa G. Health inequalities between lone and couple mothers and policy under different welfare regimes - The example of Italy, Sweden and Britain. Social Science & Medicine. 2010;70(6):912-20.
13. Bambra C, Eikemo TA. Welfare state regimes, unemployment and health: a comparative study of the relationship between unemployment and self-reported health in 23 European countries. J Epidemiol Commun H. 2009;63(2):92-8.
14. Bambra C, Pope D. What are the effects of anti-discriminatory legislation on socioeconomic inequalities in the employment consequences of ill health and disability? J Epidemiol Commun H. 2007;61(5):421-6.
15. van der Wel KA, Dahl E, Thielen K. Social inequalities in "sickness": does welfare state regime type make a difference? A multilevel analysis of men and women in 26 European countries. Int J Health Serv. 2012;42(2):235-55.
16. van der Wel KA, Dahl E, Thielen K. Social inequalities in 'sickness': European welfare states and non-employment among the chronically ill. Social Science & Medicine. 2011;73(11):1608-17.
17. Reeves A, Karanikolos M, Mackenbach J, Mckee M, Stuckler D. Do employment protection policies reduce the relative disadvantage in the labour market experienced by unhealthy people? A natural experiment created by the great recession in Europe. Soc Sci Med. 2014;121:98-108.
18. Dahl E, Fritzell J, Lahelma E, Martikainen J, Kunst A, Mackenbach JP. Welfare state regimes and health inequalities. In: Siegrist J, Marmot M, editors. Social Inequalities in Health: New evidence and policy implications. Oxford: Oxford University Press; 2006. p. 194-217.
19. Minton JW, Pickett KE, Dorling D. Health, employment, and economic change, 1973-2009: repeated cross sectional study. BMJ. 2012;344:e2316.

For peer review only - http://bmjopen.bmj.com/site/about/guidelines.xhtml

20. Whitehead M, Burstrom B, Dahl E, Clayton S, Diderichson F, Holland P, et al. Helping chronically ill or disabled people into work: what can we learn from international comparative analyses? Final report to the Department of Health. York: Public Health Research Consortium, 2009.
21. Gesis. GML: EU-SILC: GESIS; 2017 [Available from: https://www.gesis.org/gml/european-microdata/eu-silc/.
22. Barros AJ, Hirakata VN. Alternatives for logistic regression in cross-sectional studies: an empirical comparison of models that directly estimate the prevalence ratio. BMC Med Res Methodol. 2003;3:21.
23. Lee J. Odds Ratio or Relative Risk for Cross-Sectional Data. International Journal of Epidemiology. 1994;23(1):201-3.
24. Eikemo TA, Hoffmann R, Kulik MC, Kulhanova I, Toch-Marquardt M, Menvielle G, et al. How can inequalities in mortality be reduced? A quantitative analysis of 6 risk factors in 21 European populations. PLoS One. 2014;9(11):e110952.
25. OECD. How inequality compounds over the life course. Paris: OECD Publishing, 2017.
26. Maki N, Martikainen P, Eikemo T, Menvielle G, Lundberg O, Ostergren O, et al. Educational differences in disability-free life expectancy: a comparative study of long-standing activity limitation in eight European countries. Soc Sci Med. 2013;94:1-8.
27. Cambois E, Sole-Auro A, Bronnum-Hansen H, Egidi V, Jagger C, Jeune B, et al. Educational differentials in disability vary across and within welfare regimes: a comparison of 26 European countries in 2009. J Epidemiol Community Health. 2016;70(4):331-8.
28. Bambra C. Yesterday once more? Unemployment and health in the 21st century. J Epidemiol Commun H. 2010;64(3):213-5.
29. Burgard SA, Lin KY. Bad Jobs, Bad Health? How Work and Working Conditions Contribute to Health Disparities. Am Behav Sci. 2013;57(8):1105-27.
30. Leinonen T, Pietilainen O, Laaksonen M, Rahkonen O, Lahelma E, Martikainen P. Occupational social class and disability retirement among municipal employees--the contribution of health behaviors and working conditions. Scand J Work Environ Health. 2011;37(6):464-72.
31. Robroek SJ, Rongen A, Arts CH, Otten FW, Burdorf A, Schuring M. Educational Inequalities in Exit from Paid Employment among Dutch Workers: The Influence of Health, Lifestyle and Work. PLoS One. 2015;10(8):e0134867.
32. Leijten FR, de Wind A, van den Heuvel SG, Ybema JF, van der Beek AJ, Robroek SJ, et al. The influence of chronic health problems and work-related factors on loss of paid employment among older workers. J Epidemiol Community Health. 2015;69(11):1058-65.
33. Niedhammer I, Chastang JF, David S, Kelleher C. The contribution of occupational factors to social inequalities in health: findings from the national French SUMER survey. Soc Sci Med. 2008;67(11):1870-81.
34. Navarro V, Shi L. The political context of social inequalities and health. Soc Sci Med. 2001;52(3):481-91.
35. Bambra C. Health inequalities and welfare state regimes: theoretical insights on a public health 'puzzle'. J Epidemiol Community Health. 2011;65(9):740-5.
36. Pirani E, Salvini S. Is temporary employment damaging to health? A longitudinal study on Italian workers. Soc Sci Med. 2015;124:121-31.
37. Benach J, Puig-Barrachina V, Vives A, Tarafa G, Muntaner C. The challenge of monitoring employment-related health inequalities. J Epidemiol Community Health. 2012;66(12):1085-7.
38. Heggebo K, Dahl E. Unemployment and health selection in diverging economic conditions: Compositional changes? Evidence from 28 European countries. Int J Equity Health. 2015;14:121.
39. Bartley M, Sacker A, Clarke P. Employment status, employment conditions, and limiting illness: prospective evidence from the British household panel survey 1991-2001. J Epidemiol Community Health. 2004;58(6):501-6.
40. Bockerman P, Ilmakunnas P. Unemployment and self-assessed health: evidence from panel data. Health Econ. 2009;18(2):161-79.

For peer review only - http://bmjopen.bmj.com/site/about/guidelines.xhtml

41. Heistaro S, Jousilahti P, Lahelma E, Vartiainen E, Puska P. Self rated health and mortality: a long term prospective study in eastern Finland. J Epidemiol Community Health. 2001;55(4):227-32.
42. Jurges H. True health vs response styles: exploring cross-country differences in self-reported health. Health Econ. 2007;16(2):163-78.

For peer review only

For peer review only - http://bmjopen.bmj.com/site/about/guidelines.xhtml

Table 1 | Characteristics of the Study on Income and Living Conditions (EU-SILC) in 2014 by European region – 30-59 year olds

		Nordic (DK,FI, SE, NO, IS)	Western (DE, AT, NL, BE, LU, FR)	Anglo-Saxon (IE, UK)	Southern (CY, ES, IT, EL, PT)	Eastern (EE, LT, LV, CZ, SK, PL, HU, SI)
		n= 15,711 (%)	n=41,624 (%)	n=13,995 (%)	n= 52,961 (%)	n= 55,433 (%)
Sex	Female	50	53	53	52	54
Education	High	46	36	44	26	25
	Intermediate	42	47	29	36	64
	Low	13	17	28	38	11
Age	30-39	27	27	33	28	29
	40-49	35	36	35	37	33
	50-59	38	37	33	34	38
Employment Status	Employed	84	80	74	67	76
	Unemployed	5	6	7	16	9
	Economically inactive	3	7	10	11	6
	Retired	0	2	2	3	3
	Disabled	5	4	6	2	6
	Other	2	0	1	0	0
Chronic illness	Yes	33	30	27	21	29

For peer review only

Table 2 | Absolute differences in labour force participation between persons with and without a chronic illness by educational level in European regions in 2014

Region in Europe	Absolute difference (%) (95% CI)		
	Within low education	Within intermediate education	Within high education
**Male**			
Nordic	32.9% (28.9-36.8)	18.6% (16.4-20.9)	11.1% (8.5-13.7)
Continental	26.1% (23.6-28.5)	17.2% (15.7-18.6)	8.2% (6.4-10.0)
Anglo-Saxon	35.3% (31.8-38.7)	17.1% (13.1-21.1)	13.4% (9.9-17.0)
Southern	21.5% (19.7-23.3)	11.2% (8.9-13.5)	5.0% (2.2-7.9)
Eastern	33.5% (30.8-36.3)	25.5% (24.2-26.7)	9.8% (7.2-12.4)
**Female**			
Nordic	31.0% (26.2-35.7)	20.1% (17.3-22.9)	9.6% (7.1-12.0)
Continental	20.4% (17.7-23.0)	15.2% (13.5-16.9)	9.4% (7.3-11.6)
Anglo-Saxon	29.8% (25.8-33.8)	22.5% (18.5-26.6)	15.8% (12.2-19.4)
Southern	10.0% (8.0-11.9)	6.5% (4.0-8.9)	5.8% (2.9-8.6)
Eastern	23.7% (20.7-26.6)	20.6% (19.3-22.0)	6.4% (4.2-8.6)

For peer review only

Table 3 | Relative differences in labour force participation between persons with and without a chronic illness by educational level in European regions in 2014

Region in Europe	Relative difference (PR) (95% CI)		
	Within low education	Within intermediate education	Within high education
**Male**			
Nordic	1.61 (1.46-1.77)	1.25 (1.21-1.30)	1.13 (1.10-1.17)
Continental	1.49 (1.40-1.58)	1.24 (1.21-1.27)	1.09 (1.07-1.12)
Anglo-Saxon	1.79 (1.64-1.95)	1.25 (1.17-1.33)	1.17 (1.12-1.23)
Southern	1.43 (1.38-1.49)	1.16 (1.12-1.20)	1.06 (1.03-1.09)
Eastern	1.94 (1.78-2.11)	1.42 (1.39-1.45)	1.11 (1.08-1.15)
**Female**			
Nordic	1.65 (1.48-1.84)	1.30 (1.24-1.36)	1.12 (1.09-1.15)
Continental	1.48 (1.39-1.58)	1.24 (1.20-1.27)	1.12 (1.09-1.15)
Anglo-Saxon	1.89 (1.70-2.11)	1.44 (1.33-1.57)	1.23 (1.17-1.30)
Southern	1.28 (1.21-1.34)	1.11 (1.07-1.16)	1.08 (1.04-1.12)
Eastern	1.76 (1.62-1.92)	1.37 (1.33-1.40)	1.08 (1.05-1.10)

For peer review only

For peer review only - http://bmjopen.bmj.com/site/about/guidelines.xhtml

Table 4 | Trends in absolute health-related inequalities in labour force participation in European regions over the period 2005-2014

Region in Europe	Trend*		
	Low education	Intermediate education	High education
**Male**			
Nordic	0.009 (0.004-0.014)	0.001 (-0.002-0.003)	0.001 (-0.003-0.005)
Continental	0.003 (-0.001-0.007)	0.001 (0.000-0.003)	-0.002 (-0.005-0.000)
Anglo-Saxon	-0.003 (-0.013-0.007)	0.005 (-0.002-0.012)	0.003 (-0.002-0.007)
Southern	**-0.005 (-0.009--0.002)**	**-0.003 (-0.006-0.001)**	**-0.006 (-0.009--0.001)**
Eastern	0.002 (-0.004-0.009)	-0.001 (-0.004-0.002)	0.000 (-0.003-0.003)
**Female**			
Nordic	0.006 (-0.003-0.015)	-0.001 (-0.005-0.003)	-0.002 (-0.007-0.002)
Continental	**0.008 (0.004-0.011)**	**0.004 (0.003-0.006)**	0.002 (-0.002-0.005)
Anglo-Saxon	**0.006 (0.000-0.013)**	**0.008 (0.002-0.014)**	0.002 (-0.005-0.008)
Southern	-0.001 (-0.004-0.002)	0.001 (-0.003-0.005)	-0.003 (-0.008-0.003)
Eastern	**0.004 (0.000-0.008)**	0.002 (-0.001-0.004)	**-0.003 (-0.005-0.000)**

* Trend describes the widening (positive value) or narrowing (negative value) of the absolute difference of proportion in paid employment between participants with and without a chronic illness

For peer review only - http://bmjopen.bmj.com/site/about/guidelines.xhtml

Table 5 | Trends in relative health-related inequalities in labour force participation in European regions over the period 2005-2014

Region in Europe	Trend*		
	Low education	Intermediate education	High education
**Male**			
Nordic	**0.026 (0.012-0.040)**	0.002 (-0.003-0.007)	0.001 (-0.004-0.006)
Continental	**0.010 (0.001-0.020)**	0.002 (-0.001-0.005)	**-0.003 (-0.007-0.000)**
Anglo-Saxon	0.014 (-0.024-0.053)	0.014 (-0.001-0.029)	0.005 (-0.002-0.012)
Southern	-0.001 (-0.007-0.004)	-0.002 (-0.007-0.002)	**-0.006 (-0.011--0.001)**
Eastern	0.013 (-0.008-0.035)	-0.004 (-0.010-0.002)	0.000 (-0.004-0.004)
**Female**			
Nordic	0.028 (-0.004-0.060)	-0.001 (-0.008-0.006)	-0.003 (-0.009-0.002)
Continental	**0.015 (0.002-0.028)**	**0.004 (0.001-0.007)**	0.001 (-0.004-0.007)
Anglo-Saxon	0.037 (-0.003-0.077)	**0.020 (0.006-0.034)**	0.003 (-0.007-0.013)
Southern	-0.007 (-0.016-0.001)	0.003 (-0.004-0.011)	-0.003 (-0.010-0.005)
Eastern	0.010 (-0.011-0.031)	0.000 (-0.007-0.006)	-0.002 (-0.005-0.002)

* Trend describes the widening (positive value) or narrowing (negative value) of the relative difference of prevalence ratios in paid employment between participants with and without a chronic illness

For peer review only

Acknowledgement: We kindly acknowledge the help of the Microdata Access team from Eurostat for provision of the EU-SILC datasets to our research entity (2014/044/NL).

Contributorship: JLDS prepared the data, conducted the analysis, and drafted and revised the paper. MS and KMOH participated in the analysis and commented on the paper. AB conceptualised the study, participated in the analysis and commented on the paper. He is the guarantor. All authors approved the final version. We kindly thank the WORKLONG Impact Group, in particular Lars Andersen, professor at the Danish National Research Centre for the Working Environment, and Manuel Flores, economist at the Directorate for Employment, Labour and Social Affairs at the Organisation for Economic Co-operation and Development, for their comments on the draft of the paper.

Funding: This work was conceived with financial support from award no. 208060001 by ZonMW within the Joint Programming Initiative More Years Better Lives (WORKLONG project) framework. Additional financial support was received through EIT Health. The sponsor had no role in the study design; in the collection, analysis, and interpretation of data; in the writing of the report; and in the decision to submit the article for publication.

Competing interests: All authors have completed the ICMJE uniform disclosure form at www.icmje.org/coi_disclosure.pdf and declare: financial support from the Joint Programming Initiative More Years Better Lives for the submitted work: no financial relationships with any organisations that might have an interest in the submitted work in the previous three years; no other relationships or activities that could appear to have influenced the submitted work.

Ethical approval: Not required.

Data sharing: No additional data available.

The lead author (AB) affirms that the manuscript is an honest, accurate, and transparent account of the study being reported; that no important aspects of the study have been omitted; and that any discrepancies from the study as planned (and, if relevant, registered) have been explained.

This is an Open Access article distributed in accordance with the Creative Commons Attribution Non Commercial (CC BY-NC 4.0) license, which permits others to distribute, remix, adapt, build upon this work non-commercially, and license their derivative works on different terms, provided the original work is properly cited and the use is noncommercial. See: https://creativecommons.org/licenses/by-nc/4.0/.

For peer review only

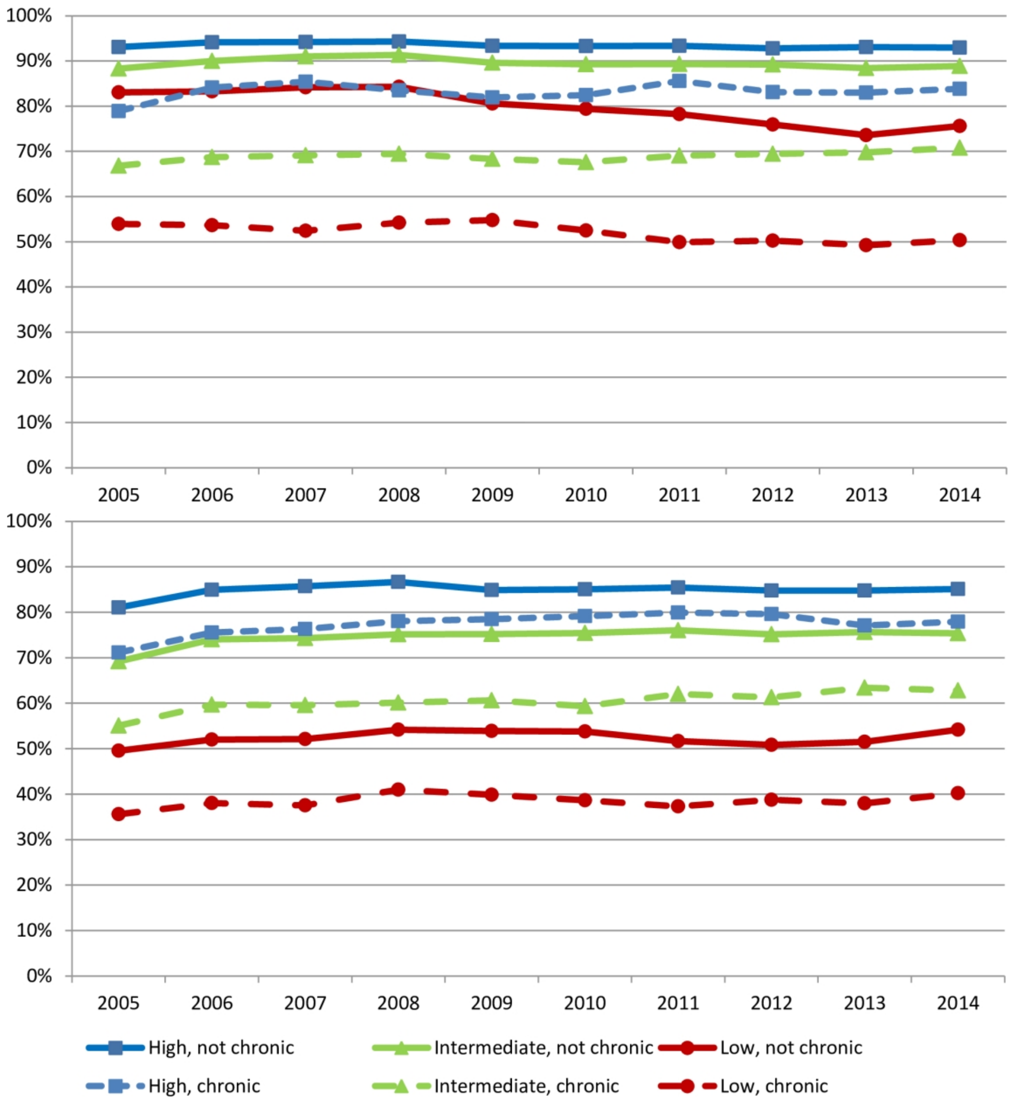

Figure 1 Labour force participation with and without chronic illness by educational level – men (above) and women (below) in 26 European countries (2005-2014)

167x182mm (300 x 300 DPI)

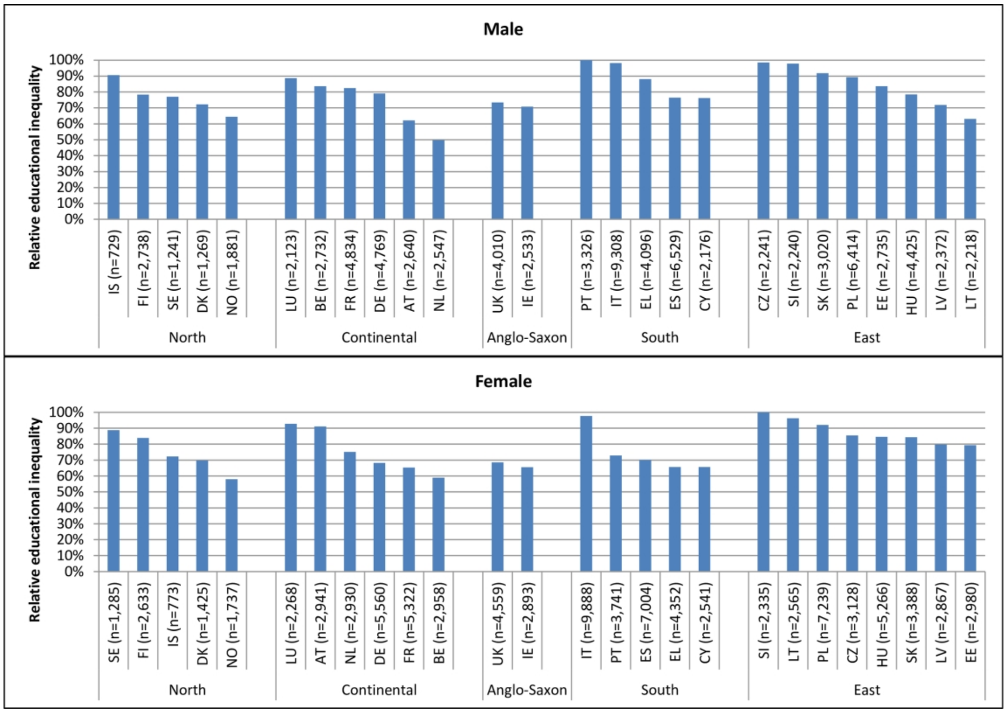

Figure 2 Relative inequality in labour force participation among persons with a chronic illness comparing low educated persons with high educated persons

190x134mm (300 x 300 DPI)

STROBE Statement—checklist of items that should be included in reports of observational studies

	Item No	Recommendation	Reported on Page No.
**Title and abstract**	1	(*a*) Indicate the study's design with a commonly used term in the title or the abstract	1, 3
		(*b*) Provide in the abstract an informative and balanced summary of what was done and what was found	3
**Introduction**			
Background/rationale	2	Explain the scientific background and rationale for the investigation being reported	4
Objectives	3	State specific objectives, including any prespecified hypotheses	4,5
**Methods**			
Study design	4	Present key elements of study design early in the paper	6-8
Setting	5	Describe the setting, locations, and relevant dates, including periods of recruitment, exposure, follow-up, and data collection	6
Participants	6	(*a*) *Cohort study*—Give the eligibility criteria, and the sources and methods of selection of participants. Describe methods of follow-up	n.a.
		Case-control study—Give the eligibility criteria, and the sources and methods of case ascertainment and control selection. Give the rationale for the choice of cases and controls	n.a.
		Cross-sectional study—Give the eligibility criteria, and the sources and methods of selection of participants	6
		(*b*) *Cohort study*—For matched studies, give matching criteria and number of exposed and unexposed	n.a.
		Case-control study—For matched studies, give matching criteria and the number of controls per case	n.a.
Variables	7	Clearly define all outcomes, exposures, predictors, potential confounders, and effect modifiers. Give diagnostic criteria, if applicable	6
Data sources/ measurement	8*	For each variable of interest, give sources of data and details of methods of assessment (measurement). Describe comparability of assessment methods if there is more than one group	6
Bias	9	Describe any efforts to address potential sources of bias	6
Study size	10	Explain how the study size was arrived at	6
Quantitative variables	11	Explain how quantitative variables were handled in the analyses. If applicable, describe which groupings were chosen and why	6
Statistical methods	12	(*a*) Describe all statistical methods, including those used to control for confounding	7-8
		(*b*) Describe any methods used to examine subgroups and interactions	7-8
		(*c*) Explain how missing data were addressed	6
		(*d*) *Cohort study*—If applicable, explain how loss to follow-up was addressed	n.a.
		Case-control study—If applicable, explain how matching of cases and controls was addressed	n.a.
		Cross-sectional study—If applicable, describe analytical methods	n.a.

For peer review only

	taking account of sampling strategy	
	(*e*) Describe any sensitivity analyses	n.a.

Continued on next page

For peer review only

For peer review only - http://bmjopen.bmj.com/site/about/guidelines.xhtml

Results			Reported on Page No.
Participants	13*	(a) Report numbers of individuals at each stage of study—eg numbers potentially eligible, examined for eligibility, confirmed eligible, included in the study, completing follow-up, and analysed	9, 17
		(b) Give reasons for non-participation at each stage	n.a.
		(c) Consider use of a flow diagram	n.a.
Descriptive data	14*	(a) Give characteristics of study participants (eg demographic, clinical, social) and information on exposures and potential confounders	9, 17
		(b) Indicate number of participants with missing data for each variable of interest	n.a.
		(c) *Cohort study*—Summarise follow-up time (eg, average and total amount)	n.a
Outcome data	15*	*Cohort study*—Report numbers of outcome events or summary measures over time	n.a.
		Case-control study—Report numbers in each exposure category, or summary measures of exposure	*n.a.*
		Cross-sectional study—Report numbers of outcome events or summary measures	9,17
Main results	16	(*a*) Give unadjusted estimates and, if applicable, confounder-adjusted estimates and their precision (eg, 95% confidence interval). Make clear which confounders were adjusted for and why they were included	9, 18-21
		(*b*) Report category boundaries when continuous variables were categorized	9, 18-21
		(*c*) If relevant, consider translating estimates of relative risk into absolute risk for a meaningful time period	n.a.
Other analyses	17	Report other analyses done—eg analyses of subgroups and interactions, and sensitivity analyses	9,10,25
**Discussion**			
Key results	18	Summarise key results with reference to study objectives	11-12
Limitations	19	Discuss limitations of the study, taking into account sources of potential bias or imprecision. Discuss both direction and magnitude of any potential bias	12
Interpretation	20	Give a cautious overall interpretation of results considering objectives, limitations, multiplicity of analyses, results from similar studies, and other relevant evidence	11-12
Generalisability	21	Discuss the generalisability (external validity) of the study results	12
**Other information**			
Funding	22	Give the source of funding and the role of the funders for the present study and, if applicable, for the original study on which the present article is based	22

*Give information separately for cases and controls in case-control studies and, if applicable, for exposed and unexposed groups in cohort and cross-sectional studies.

Note: An Explanation and Elaboration article discusses each checklist item and gives methodological background and published examples of transparent reporting. The STROBE checklist is best used in conjunction with this article (freely available on the Web sites of PLoS Medicine at http://www.plosmedicine.org/, Annals of Internal Medicine at http://www.annals.org/, and Epidemiology at http://www.epidem.com/). Information on the STROBE Initiative is available at www.strobe-statement.org.

For peer review only

For peer review only - http://bmjopen.bmj.com/site/about/guidelines.xhtml

BMJ Open

Health-related educational inequalities in paid employment across 26 European countries in 2005-2014: a repeated cross-sectional study

Journal:	*BMJ Open*
Manuscript ID	bmjopen-2018-024823.R1
Article Type:	Research
Date Submitted by the Author:	22-Sep-2018
Complete List of Authors:	Schram, Jolinda; Erasmus MC, Department of Public Health<br>Schuring, Merel; Erasmus MC<br>Oude Hengel, Karen; Erasmus MC; Netherlands Organization for Applied Scrientific Research TNO<br>Burdorf, Alex; Erasmus MC, Department of Public Health
**Primary Subject Heading**:	Public health
Secondary Subject Heading:	Global health
Keywords:	Trend analysis, Social inequalities, Chronic illness, Employment

SCHOLARONE™
Manuscripts

Title **Health-related educational inequalities in paid employment across 26 European countries in 2005-2014: a repeated cross-sectional study**

Authors' names Jolinda LD Schram[1], Merel Schuring[1], Karen M Oude Hengel[1,2], Alex Burdorf[1]

Corresponding author Correspondence to: Merel Schuring, Erasmus University Medical Center, Department of Public Health, P.O. Box 2040, 3000 CA Rotterdam, The Netherlands, m.schuring@erasmusmc.nl , +31107038993

Affiliation [1] Erasmus University Medical Center, Department of Public Health, P.O. Box 2040, 3000 CA Rotterdam, The Netherlands.
[2] Netherlands Organization for Applied Scientific Research TNO, P.O. Box 3005, 2301 DA Leiden, The Netherlands.

Key words Social inequalities, Employment, Chronic illness, Trend analysis

Word count 3,933

For peer review only

ABSTRACT

Objective: The study investigates trends in health-related inequalities in paid employment among men and women in different educational groups in 26 countries in five European regions.

Design: Individual-level analysis of repeated cross-sectional annual data (2005-2014) from the EU Statistics on Income and Living Conditions (EU-SILC).

Setting: 26 European countries in five European regions.

Participants: 1,844,915 individuals age 30-59 years were selected with information on work status, chronic illness, educational background, age, and gender.

Outcome measures: Absolute differences were expressed by absolute differences in proportion in paid employment between participants with and without a chronic illness, using linear regression. Relative differences were expressed by prevalence ratios in paid employment, using a Cox proportional hazard model. Linear regression was used to examine the trends of inequalities.

Results: Participants with a chronic illness had consistently lower labour force participation than those without illnesses. Educational inequalities were substantial with absolute differences larger within lower educated (men 21-35%, women 10-31%) than within higher educated (men 5-13%, women 6-16%). Relative differences showed that low educated men with a chronic illness were 1.4-1.9 times (women 1.3-1.8 times) more likely to be out of paid employment than low educated persons without a chronic illness, whereas this was 1.1-1.2 among high educated men and women. In the Nordic, Anglo-Saxon and Eastern regions these health-related educational inequalities in paid employment were more pronounced than in the Continental and Southern region. For most regions, absolute health-related educational inequalities in paid employment were generally constant, whereas relative inequalities increased, especially among low educated persons.

Conclusions: Men and women with a chronic illness have considerable less access to the labour market than their healthy colleagues, especially among lower educated persons. This exclusion from paid employment will increase health inequalities.

For peer review only - http://bmjopen.bmj.com/site/about/guidelines.xhtml

Strengths and limitations of the study

- This is the first study to present information on trends in health-related educational inequalities in paid employment among persons in different European regions
- A strength of the current study is the use of cross sectional data from a large number of European countries and large number of individuals for which the outcomes are harmonized (1,844,915 individuals in 26 countries).
- The effects of having a chronic illness on being employed (selection) and unemployment causing ill health such as chronic illness (causation), cannot be distinguished by using cross-sectional data.
- Although the outcomes of EU-SILC are harmonized, comparisons in self-rated health measures between different cultures need to be made with caution.

For peer review only

For peer review only - http://bmjopen.bmj.com/site/about/guidelines.xhtml

Introduction

Life expectancy continues to increase and will ensure that the old-age dependency ratio, the number of elderly people as a share of those of working age, will rise steadily from 29.4 in 2016 to 50.3 by 2050 in Europe (1, 2). In response, many countries have raised the statutory retirement age (3). The challenge is ensuring that those of working age are actually participating in the labour force (4). Health-related exclusion from the labour market is present in many countries (5, 6), whereby workers with a chronic illness are at increased risk of becoming disabled or unemployed. Leaving paid employment may further impact health negatively (7).

Studies have shown that the presence of a chronic disease is an important barrier for entering and maintaining paid employment (5). Educational inequalities in morbidity, such as chronic health conditions, are well documented across European countries (8). Some studies have suggested that the effect of chronic illness on maintaining paid employment differs by socioeconomic position. Bartley and Owen reported that workers with a chronic disease in lower socioeconomic position had more difficulties staying in paid employment than those with a higher socioeconomic position during times of economic crisis (9). In Sweden and the United Kingdom labour market attachment for those with a chronic illness is reduced, with the effect being substantially stronger in the United Kingdom than in Sweden (10, 11).

The differential effect of socioeconomic position on the association between chronic illness and paid employment may be modified by national social and economic conditions, such as labour market policies (12). European countries can be categorized in five regions: the Nordic, Continental, Anglo-Saxon, Southern, and Eastern regions (13, 14). This typology reflects general differences in employment protection and income security. Non-employment rates for persons with a longstanding illness as well as a low educational level were found particularly high in the Anglo-Saxon and Eastern regions, compared to the Nordic region (15). The poor position of disabled people was suggested to be partly counteracted by employment protection (16), although inequalities in health in itself were not consistently smaller in the Nordic region (17).

Few studies have documented the trends over time in socioeconomic differences in labour force participation between persons with and without a chronic illness. A repeated cross sectional study over 25 years in Britain showed that within the lower occupational class persons with a limiting illness had greater and more rapidly widening gaps in labour force participation than in the higher occupational class (18). A comparison between the Nordic countries and the United Kingdom showed that, since the mid-1990s, there has been a deterioration in paid employment among persons with limiting long standing illness with a low education, even when the economy was doing well (19).

Limited information exists on trends in health-related educational inequalities in paid employment among persons in different European regions, since most studies have focused on a single country or compared a selected group of countries. Therefore, the aims of this paper are (i) to examine absolute and relative differences in paid employment between persons with and without a chronic illness within each educational group in five European regions and (ii) to describe trends in socioeconomic differences in paid employment between people with and without a chronic illness within European countries.

For peer review only - http://bmjopen.bmj.com/site/about/guidelines.xhtml

Methods

Data

A repeated cross-sectional study was conducted with the EU-SILC survey (European Union Statistics on Income and Living Conditions). The EU-SILC is a survey questionnaire designed to provide comparable information across the EU and cross-sectional data from 2005 to 2014 was used from 26 countries that participated in EU-SILC since 2005 (Denmark, Finland, Iceland, Norway, Sweden, Austria, Belgium, Germany, France, Luxembourg, The Netherlands, Cyprus, Greece, Italy, Portugal, Spain, Ireland, United Kingdom, Czech Republic, Estonia, Hungary, Latvia, Lithuania, Poland, Slovakia and Slovenia). Detailed information on the EU-SILC study design can be found elsewhere (20).

Respondents were selected with information on work status, chronic illness, educational background, age, and gender. Our study comprises 179,724 individuals aged 30–59 years in 2014. For the trend analysis 1,844,915 individuals were selected aged 30-59 years between 2005-2014. The age limit of 59 years was applied because a number of countries have an official retirement age of 60, thereby greatly reducing the proportion of employed persons after 60 years. The age limit of 30 years was used to exclude potential confounding due to different educational pathways between those with a chronic illness and those without.

Employment status

Employment status was classified into six mutually exclusive categories: Employed (working full or part-time, self-employed full or part-time), unemployed, economically inactive (fulfilling domestic tasks and care responsibilities, and other inactivity), retired, disabled and other (student, in compulsory military community or service). For the main analysis a dichotomized variable was used: paid employment or outside of paid employment (all other options).

Chronic illness

The question *Do you have any longstanding illness or health problem?* was used to distinguish between people with or without a chronic illness.

Sociodemographic variables

Three sociodemographic variables were used in the study: age, gender, and education. Age was categorized into three 10-year age groups: 30-39, 40-49 and 50-59 years. Educational level was recorded as the highest level of education completed by the respondent. We coded education according to the 1997 International Standard Classification of Education (ISCED-97) and categorized as low (0-2: pre-primary, primary and lower secondary education), intermediate (3-4: upper secondary education/post-secondary non tertiary) and high (5-6: tertiary education).

Regions

The European countries were categorized into five different regions, based on relative comparability of welfare state regimes and geography (13). The Nordic region included Denmark, Finland, Iceland, Norway, and Sweden. The Continental region included Austria, Belgium, Germany, France, Luxembourg, and The Netherlands. The Southern region included Cyprus, Greece, Italy, Portugal, and Spain. The Anglo-Saxon countries included Ireland and the United Kingdom. The Eastern region included the Czech Republic, Estonia, Hungary, Latvia, Lithuania, Poland, Slovakia, and Slovenia.

For peer review only - http://bmjopen.bmj.com/site/about/guidelines.xhtml

Statistical analysis

The statistical analysis focuses on both absolute and relative differences in labour force participation among those with and without a chronic disease across different educational groups. The absolute difference depicts the reduction of being in paid employment among persons with a chronic illness and is of special interest as it presents the impact on the total population in %point employment rate. The relative difference presents the prevalence ratio as a measure of the strength of the association between having a chronic illness and being out of paid employment. Absolute and relative inequalities may differ because levels of labour force participation are generally higher in higher educated groups.

Annual sample weights were used to estimate the prevalence of labour market participation that are representative for the countries included in this study. For each region the association of having a chronic illness with labour force participation within each educational group was analysed using two different models. A linear regression model was used to calculate absolute differences in labour force participation between persons with and without a chronic illness. Cox regression analysis with a robust estimate was applied to calculate relative differences. Cox regression analysis was applied instead of logistic regression. This model will more accurately describe the association of relative risk between being in paid employment and having a chronic illness than odds ratios since the odds ratio will overestimate the prevalence ratio due to the high prevalence of being in paid employment (21, 22).

The linear regression model to calculate absolute difference can be written as:

$$y = \alpha + \beta_1 low + \beta_2 intermediate + \beta_3 low\_chronic + \beta_4 intermediate\_chronic + \beta_5 high\_chronic + \beta_6 age$$

where *y* is a dichotomous variable for being employed; α is an intercept, two dummy variables to compare low and intermediate education with high education, and three additional dummy variables to compare those with a chronic illness to those without in each education group. Adjustment for age was done by default.

The Cox proportional hazard model to calculate relative differences can be written as:

$$h(t,x) = h_0(t)\exp\left(\sum_{k=1}^{p} \beta_1 low + \beta_2 intermediate + \beta_3 low\_chronic + \beta_4 intermediate\_chronic + \beta_5 high\_chronic + \beta_6 age\right)$$

where *h(t,…)* is the likelihood at time *t* that a person is not employed, two dummy variables for low and intermediate education to compare to high education, and three dummy variables to compare those with a chronic illness in each education group. Adjustment for age was done by default.

The time period for all persons was set at a constant level (value 1) to estimate the prevalence ratio (PR) (21, 23). In all analyses, variables were recoded so a PR above 1 indicates an increased likelihood of being out of paid employment with corresponding 95% confidence intervals (95% CI). The associations were adjusted for age and stratified by region and sex. Significance was determined by $P < 0.05$.

Trend analysis in the five regions

For peer review only - http://bmjopen.bmj.com/site/about/guidelines.xhtml

For determining the trends in absolute and relative health-related inequalities in being in paid employment over 10 years in each region, we used linear regression analyses with the absolute gap and prevalence ratios in each educational group as dependent variable in each survey year. The analysis was stratified by region and gender. The year of data collection was used as the independent variable.

Health-related educational inequalities in paid employment

In order to estimate educational inequalities in the relative differences in labour force participation among persons with a chronic illness a counterfactual scenario was introduced. In this scenario it is assumed that the relative difference in labour force participation between those with a chronic illness and those without in the lower educational group can be reduced to the relative difference observed in the higher educational group (24). The estimation of the excess relative difference in paid employment for chronic illness status in lower educated workers is a fair reflection of educational inequalities between low and high educated persons. The Cox proportional hazard model was used to estimate this excess relative difference by the formula: (bèta for relative difference in the low educated group – bèta for relative difference in the high educated group)/(bèta of the relative difference in the low educated group).

The main analysis was performed using Stata statistical software version 14 (Stata-Corp, College Station, Texas). The estimates of paid employment per country and the trend analysis were conducted in IBM SPSS statistical software version 22 (SPSS Inc, Chicago, IL, USA).

Patient involvement

Not applicable, we have used available datasets from Eurostat for secondary data analysis.

For peer review only - http://bmjopen.bmj.com/site/about/guidelines.xhtml

Results

The study population consisted of 179,724 participants of whom half were women, the largest group was intermediately educated, 75% were employed, and 27% had a chronic illness. The Eastern region had a large intermediate educated group (64%), whereas the Southern and Anglo-Saxon regions had a relatively large low educated group (28 and 38%, respectively) (Table 1). The Southern region had the lowest labour force participation, and the lowest percentage of people with a chronic illness. The Nordic region had the highest employment, but also the highest prevalence of people with a chronic illness.

Labour force participation over the period 2005-2014 among men without a chronic illness remained stable for intermediate and high educated persons, but decreased by about 5% among those with lower education (Figure 1A). Within each educational group, labour force participation among men with a chronic illness was substantially lower than among men without illness, with the largest difference among the lowest educated persons. For women the labour force participation increased slightly for most groups over the period 2005-2014 (Figure 1B). Women had a consistently lower labour force participation than men with increasing differences by lower educational level. Women with a chronic disease had substantially lower labour force participation than women without a chronic illness.

The absolute differences in labour force participation between persons with and without a chronic illness increased with lower educational level, among men and women (Table 2). The absolute gaps for men were larger than for women. For low educated persons the absolute differences in labour force participation between men with and without a chronic illness were higher in the Nordic, Anglo-Saxon and Eastern region (32.9-35.3%) than in the Continental and Southern region (21.5-26.1%). Among women a comparable pattern was observed.

A similar pattern was found for relative differences in health-related labour force participation (Table 3). Overall, low educated men with a chronic illness were 1.4 to 1.9 times more likely to be outside of paid employment than low educated men without a chronic illness, whereas this was 1.1 to 1.2 times within the high educational group. Relative differences in labour force participation among low educated men without and with a chronic illness were the highest in the Anglo-Saxon and Eastern region (1.79-1.94). For men with high education modest regional differences were found in labour force participation between those without and with a chronic illness. Comparable patterns were observed for women.

Trends in absolute differences in labour force participation showed stable inequalities for men in all educational groups in most regions (Table 4, Suppl. Table 1,2). Among men, significant narrowing of absolute health-related inequalities in paid employment was observed for all educational categories in the Southern region over the period 2005-2014. For women, significant widening of absolute health-related inequalities in paid employment were observed for low and intermediate educated women in the Continental and Anglo-Saxon region. In the Eastern region significant widening in inequalities was observed for low educated women, while narrowing inequalities were observed for high educated women.

Trends in relative differences in labour force participation were constant for high educated men and widened for low educated men (Table 5, Suppl. Table 3,4). Among men, significant widening of relative differences were observed for lower educated persons in the Nordic and Continental region of respectively 1% and 2.6% over the period 2005-2014. Significant narrowing of relative health-related inequalities in paid employment was observed for higher educated men in the Continental and Southern region (-0.3% and -0.6%). Trends for women were similar. In most regions relative health-related inequalities in paid employment were widening for low educated women (between

For peer review only - http://bmjopen.bmj.com/site/about/guidelines.xhtml

1.0% and 3.7%), although in the Southern region there was a small narrowing (-0.7%). For high educated women there was no clear trend.

Figure 2 presents the inequalities between low and high educated persons for the influence of a chronic illness on being in paid employment within each educational group. The excess relative difference among low educated persons across 26 European countries varied between 50% to 100% among men and between 58% and 100% among women when compared to high educated persons. The variation in educational inequalities in labour market position of those with a chronic disease was larger within European regions than between European regions, indicating substantial differences between countries with supposedly similar welfare regimes.

For peer review only

For peer review only - http://bmjopen.bmj.com/site/about/guidelines.xhtml

Discussion

Within Europe, persons with a chronic disease were less often in paid employment than persons without a chronic disease in the period 2005-2014. The low education group had the largest absolute and relative differences in paid employment between persons with and without a chronic illness. For men these differences were more pronounced than for women. The Nordic, Anglo-Saxon and Eastern regions had substantially higher relative and absolute differences in paid employment than in the Continental and Southern region. The relative position on the labour market for persons with a chronic illness worsened in the past 10 years among low educated persons, and remained fairly stable for high educated persons.

The risk of being outside of paid employment with a chronic illness is higher for persons with a low education than those with a high education. Previous studies have shown comparable results in specific countries (9, 11, 16, 18) or across Europe in one particular year. (25, 26) Our study expands this knowledge by demonstrating that these patterns are consistent over time and European countries, and by illustrating that health-related educational inequalities in paid employment have widened rather than narrowed in the past decade.

Men and women with a chronic illness have less access to the labour market than their healthy colleagues, especially among lower educated persons. Possible explanations for the observed educational inequality relate to severity of chronic illnesses and quality of jobs. In this study chronic illness could not be differentiated by diagnosis and severity. It may be hypothesised that lower educated workers more often suffer from chronic diseases with functional limitations than high educated workers (27, 28). Having a chronic illness and functional limitations follows a social gradient in our data (high educated 49%, low educated 73%). The education inequality may also be explained by differences in strenuous work and other job characteristics that may interfere with health problems (29, 30).

A few studies have demonstrated that strenuous work conditions hamper workers with a chronic illness to remain in paid employment. Among Finnish workers physical work load strongly mediated the association between musculoskeletal disorders and exit from paid employment (31). A Dutch study attributed 12% of educational differences in disability benefits to work characteristics (32). Another Dutch study showed that high autonomy, high social support and low psychosocial job strongly moderated the association between chronic health conditions and exit through disability benefits (33). Lower socio-economic jobs more often have unfavourable work characteristics (34). Therefore, the ability to remain in paid employment is reduced for low educated persons with a chronic illness.

Health-related absolute and relative differences in paid employment across educational groups were consistently lower in the Southern region than in other European regions. A few studies (12, 18, 25) have noted larger absolute and relative differences in labour force participation between persons with and without a chronic illness in regions with low employment protection compared to regions with high employment protection. Our results in the Southern region, which is characterised by high employment protection, corroborates these previous studies. However, the educational inequalities in paid employment depicted in figure 2 showed a larger variation between countries in the same region than between different regions. This suggests that other social, legal and economic conditions, such as availability of social benefits, also play a role in being in paid employment of people with a chronic illness. More studies are necessary to evaluate the influence of various national indicators of social, cultural and economic conditions on the variation in health-related educational inequalities in paid employment.

For peer review only - http://bmjopen.bmj.com/site/about/guidelines.xhtml

The absolute and relative disadvantages in labour force participation among those with a chronic disease were more pronounced for men than for women in most welfare states. In the Southern welfare state regime the absolute and relative differences were smaller for women, but in the Nordic welfare state regime the two groups showed similar patterns. These regional differences between gender may partly be explained by the full-employment policies of the Nordic welfare state regime, that facilitate the integration of women into the labour force by providing family supportive services such as child care (35). The high labour force participation of women in Nordic welfare states can contribute to higher relative differences between those with and without a chronic illness compared to the Southern region. This is in line with previous studies stating that in terms of socioeconomic inequalities the relative inequalities do not appear to be smallest in more redistributive countries such as the Scandinavian welfare state, but that the absolute health of all social classes is often better (36).

The trends observed in this study showed that over the period 2005 to 2014 educational inequalities in paid employment of persons with a chronic illness have widened among low educated persons. In the recent decades precarious employment, such as temporary contracts and part-time work, have become more common in Europe (37, 38). Further research is necessary to evaluate whether the increase in precarious employment could be an explanation for increasing educational inequalities in paid employment.

The trend analysis might be influenced by the differential effect of the economic crisis across regions. The Continental and Eastern region have had an increase in persons in paid employment over the period 2005-2014, while the Southern region had a decrease of persons in paid employment due to the economic crisis. In periods of high and rapid increase in unemployment rates, workers in good health will also lose their jobs and, thus, the influence of ill health on paid employment will attenuate (39). This mechanism may explain why we found a decreasing trend in the Southern region. Furthermore, health may have worsened during the economic crisis. For persons with existing health issues, this could have led to more difficulties maintaining paid employment. A recent study found an increase in absolute inequalities in health between educational groups after the recent economic crisis in the Continental and Anglo-Saxon region (40). This may explain the increasing trends we found in these regions.

Strengths and limitations

Data were derived from the European Union Statistics on Income and Living Conditions (EU-SILC). A strength of the current study is the use of cross sectional data from a large number of European countries for which the outcomes are harmonized. By utilizing Cox regression instead of logistic regression the different sample designs in EU SILC across countries will not affect the results of the statistical analysis. The repeated cross-sectional surveys present a snapshot of the labour force situation of each region for the respective years, but causal relations cannot be investigated in depth, as other studies with cohort information have done (41, 42). By using cross sectional data in this study the effects of having a chronic illness on paid employment (selection) and unemployment causing ill health such as chronic illness (causation), cannot be distinguished.

A limitation of using EU-SILC data is the variation in mode of data collection, translations, and cultural interpretation (25). Further limitations are the use of self-reported health and labour market measures which could vary by country, socioeconomic position and cultural beliefs (13). However, a number of studies have shown that self-rated measurements of health are strongly predictive for objective health measures, such as mortality (43). Comparisons in self-rated health measures between different cultures do need to be made with caution (44).

Policy implications

For peer review only - http://bmjopen.bmj.com/site/about/guidelines.xhtml

Men and women with a chronic illness have considerable less access to the labour market than their healthy colleagues, especially among lower educated persons. Their position in the labour market seems to have worsened rather than improved in the past decade. Persons with a chronic illness without a paid job are at risk for further deterioration of their health and economic marginalisation. More effective policy measures are needed to reduce the gap in paid employment between those with and without a chronic illness, especially in the low education group, in all European regions.

For peer review only

For peer review only - http://bmjopen.bmj.com/site/about/guidelines.xhtml

References

1. Eurostat. Projected old-age dependency ratio: Eurostat; 2013 [updated 11 September 2017. Available from: http://ec.europa.eu/eurostat/tgm/table.do?tab=table&init=1&language=en&pcode=tsdde511&plugin=1.
2. Eurostat. Old-age-dependency ratio: Eurostat; 2017 [updated 11 September 2017. Available from: http://ec.europa.eu/eurostat/tgm/table.do?tab=table&init=1&language=en&pcode=tsdde510&plugin=1.
3. Christensen K, Doblhammer G, Rau R, Vaupel JW. Ageing populations: the challenges ahead. Lancet. 2009;374(9696):1196-208.
4. Spijker J, MacInnes J. Population ageing: the timebomb that isn't? BMJ. 2013;347:f6598.
5. van Rijn RM, Robroek SJ, Brouwer S, Burdorf A. Influence of poor health on exit from paid employment: a systematic review. Occup Environ Med. 2014;71(4):295-301.
6. Carr E, Fleischmann M, Goldberg M, Kuh D, Murray ET, Stafford M, et al. Occupational and educational inequalities in exit from employment at older ages: evidence from seven prospective cohorts. Occup Environ Med. 2018.
7. Schuring M, Robroek SJ, Lingsma HF, Burdorf A. Educational differences in trajectories of self-rated health before, during, and after entering or leaving paid employment in the European workforce. Scand J Work Environ Health. 2015;41(5):441-50.
8. Dalstra JAA, Kunst AE, Borrell C, Breeze E, Cambois E, Costa G, et al. Socioeconomic differences in the prevalence of common chronic diseases: an overview of eight European countries. International Journal of Epidemiology. 2005;34(2):316-26.
9. Bartley M, Owen C. Relation between socioeconomic status, employment, and health during economic change, 1973-93. Brit Med J. 1996;313(7055):445-9.
10. Lindholm C, Burstrom B, Diderichsen F. Class differences in the social consequences of illness? J Epidemiol Commun H. 2002;56(3):188-92.
11. Burstrom B, Holland P, Diderichsen F, Whitehead M. Winners and losers in flexible labor markets: The fate of women with chronic illness in contrasting policy environments - Sweden and Britain. International Journal of Health Services. 2003;33(2):199-217.
12. Burstrom B, Whitehead M, Clayton S, Fritzell S, Vannoni F, Costa G. Health inequalities between lone and couple mothers and policy under different welfare regimes - The example of Italy, Sweden and Britain. Social Science & Medicine. 2010;70(6):912-20.
13. Bambra C, Eikemo TA. Welfare state regimes, unemployment and health: a comparative study of the relationship between unemployment and self-reported health in 23 European countries. J Epidemiol Commun H. 2009;63(2):92-8.
14. Bambra C, Pope D. What are the effects of anti-discriminatory legislation on socioeconomic inequalities in the employment consequences of ill health and disability? J Epidemiol Commun H. 2007;61(5):421-6.
15. van der Wel KA, Dahl E, Thielen K. Social inequalities in "sickness": does welfare state regime type make a difference? A multilevel analysis of men and women in 26 European countries. Int J Health Serv. 2012;42(2):235-55.
16. Reeves A, Karanikolos M, Mackenbach J, Mckee M, Stuckler D. Do employment protection policies reduce the relative disadvantage in the labour market experienced by unhealthy people? A natural experiment created by the Great Recession in Europe. Social Science & Medicine. 2014;121:98-108.
17. Dahl E, Fritzell J, Lahelma E, Martikainen J, Kunst A, Mackenbach JP. Welfare state regimes and health inequalities. In: Siegrist J, Marmot M, editors. Social Inequalities in Health: New evidence and policy implications. Oxford: Oxford University Press; 2006. p. 194-217.
18. Minton JW, Pickett KE, Dorling D. Health, employment, and economic change, 1973-2009: repeated cross sectional study. BMJ. 2012;344:e2316.

For peer review only - http://bmjopen.bmj.com/site/about/guidelines.xhtml

19. Whitehead M, Burstrom B, Dahl E, Clayton S, Diderichson F, Holland P, et al. Helping chronically ill or disabled people into work: what can we learn from international comparative analyses? Final report to the Department of Health. York: Public Health Research Consortium, 2009.
20. Gesis. GML: EU-SILC: GESIS; 2017 [Available from: https://www.gesis.org/gml/european-microdata/eu-silc/.
21. Barros AJ, Hirakata VN. Alternatives for logistic regression in cross-sectional studies: an empirical comparison of models that directly estimate the prevalence ratio. BMC Med Res Methodol. 2003;3:21.
22. Ukoumunne OC, Forbes AB, Carlin JB, Gulliford MC. Comparison of the risk difference, risk ratio and odds ratio scales for quantifying the unadjusted intervention effect in cluster randomized trials. Stat Med. 2008;27(25):5143-55.
23. Lee J. Odds Ratio or Relative Risk for Cross-Sectional Data. International Journal of Epidemiology. 1994;23(1):201-3.
24. Eikemo TA, Hoffmann R, Kulik MC, Kulhanova I, Toch-Marquardt M, Menvielle G, et al. How can inequalities in mortality be reduced? A quantitative analysis of 6 risk factors in 21 European populations. PLoS One. 2014;9(11):e110952.
25. van der Wel KA, Dahl E, Thielen K. Social inequalities in 'sickness': European welfare states and non-employment among the chronically ill. Social Science & Medicine. 2011;73(11):1608-17.
26. OECD. How inequality compounds over the life course. Paris: OECD Publishing, 2017.
27. Maki N, Martikainen P, Eikemo T, Menvielle G, Lundberg O, Ostergren O, et al. Educational differences in disability-free life expectancy: a comparative study of long-standing activity limitation in eight European countries. Soc Sci Med. 2013;94:1-8.
28. Cambois E, Sole-Auro A, Bronnum-Hansen H, Egidi V, Jagger C, Jeune B, et al. Educational differentials in disability vary across and within welfare regimes: a comparison of 26 European countries in 2009. J Epidemiol Community Health. 2016;70(4):331-8.
29. Bambra C. Yesterday once more? Unemployment and health in the 21st century. J Epidemiol Commun H. 2010;64(3):213-5.
30. Burgard SA, Lin KY. Bad Jobs, Bad Health? How Work and Working Conditions Contribute to Health Disparities. Am Behav Sci. 2013;57(8):1105-27.
31. Leinonen T, Pietilainen O, Laaksonen M, Rahkonen O, Lahelma E, Martikainen P. Occupational social class and disability retirement among municipal employees--the contribution of health behaviors and working conditions. Scand J Work Environ Health. 2011;37(6):464-72.
32. Robroek SJ, Rongen A, Arts CH, Otten FW, Burdorf A, Schuring M. Educational Inequalities in Exit from Paid Employment among Dutch Workers: The Influence of Health, Lifestyle and Work. PLoS One. 2015;10(8):e0134867.
33. Leijten FR, de Wind A, van den Heuvel SG, Ybema JF, van der Beek AJ, Robroek SJ, et al. The influence of chronic health problems and work-related factors on loss of paid employment among older workers. J Epidemiol Community Health. 2015;69(11):1058-65.
34. Niedhammer I, Chastang JF, David S, Kelleher C. The contribution of occupational factors to social inequalities in health: findings from the national French SUMER survey. Soc Sci Med. 2008;67(11):1870-81.
35. Navarro V, Shi L. The political context of social inequalities and health. Soc Sci Med. 2001;52(3):481-91.
36. Bambra C. Health inequalities and welfare state regimes: theoretical insights on a public health 'puzzle'. J Epidemiol Community Health. 2011;65(9):740-5.
37. Pirani E, Salvini S. Is temporary employment damaging to health? A longitudinal study on Italian workers. Soc Sci Med. 2015;124:121-31.
38. Benach J, Puig-Barrachina V, Vives A, Tarafa G, Muntaner C. The challenge of monitoring employment-related health inequalities. J Epidemiol Community Health. 2012;66(12):1085-7.
39. Heggebo K, Dahl E. Unemployment and health selection in diverging economic conditions: Compositional changes? Evidence from 28 European countries. Int J Equity Health. 2015;14:121.

For peer review only - http://bmjopen.bmj.com/site/about/guidelines.xhtml

40. Leao T, Campos-Matos I, Bambra C, Russo G, Perelman J. Welfare states, the Great Recession and health: Trends in educational inequalities in self-reported health in 26 European countries. Plos One. 2018;13(2).

41. Bartley M, Sacker A, Clarke P. Employment status, employment conditions, and limiting illness: prospective evidence from the British household panel survey 1991-2001. J Epidemiol Community Health. 2004;58(6):501-6.

42. Bockerman P, Ilmakunnas P. Unemployment and self-assessed health: evidence from panel data. Health Econ. 2009;18(2):161-79.

43. Heistaro S, Jousilahti P, Lahelma E, Vartiainen E, Puska P. Self rated health and mortality: a long term prospective study in eastern Finland. J Epidemiol Community Health. 2001;55(4):227-32.

44. Jurges H. True health vs response styles: exploring cross-country differences in self-reported health. Health Econ. 2007;16(2):163-78.

For peer review only

Table 1 | Characteristics of the Study on Income and Living Conditions (EU-SILC) in 2014 by European region – 30-59 year olds

		Nordic (DK,FI, SE, NO, IS)	Western (DE, AT, NL, BE, LU, FR)	Anglo-Saxon (IE, UK)	Southern (CY, ES, IT, EL, PT)	Eastern (EE, LT, LV, CZ, SK, PL, HU, SI)
		n= 15,711 (%)	n=41,624 (%)	n=13,995 (%)	n= 52,961 (%)	n= 55,433 (%)
Sex	Female	50	53	53	52	54
Education	High	46	36	44	26	25
	Intermediate	42	47	29	36	64
	Low	13	17	28	38	11
Age	30-39	27	27	33	28	29
	40-49	35	36	35	37	33
	50-59	38	37	33	34	38
Employment Status	Employed	84	80	74	67	76
	Unemployed	5	6	7	16	9
	Economically inactive	3	7	10	11	6
	Retired	0	2	2	3	3
	Disabled	5	4	6	2	6
	Other	2	0	1	0	0
Chronic illness	Yes	33	30	27	21	29

For peer review only

Table 2 | Absolute differences in paid employment between persons with and without a chronic illness by educational level in European regions in 2014

Region in Europe	Absolute difference (%) (95% CI)		
	Within low education	Within intermediate education	Within high education
**Male**			
Nordic	32.9% (28.9-36.8)	18.6% (16.4-20.9)	11.1% (8.5-13.7)
Continental	26.1% (23.6-28.5)	17.2% (15.7-18.6)	8.2% (6.4-10.0)
Anglo-Saxon	35.3% (31.8-38.7)	17.1% (13.1-21.1)	13.4% (9.9-17.0)
Southern	21.5% (19.7-23.3)	11.2% (8.9-13.5)	5.0% (2.2-7.9)
Eastern	33.5% (30.8-36.3)	25.5% (24.2-26.7)	9.8% (7.2-12.4)
**Female**			
Nordic	31.0% (26.2-35.7)	20.1% (17.3-22.9)	9.6% (7.1-12.0)
Continental	20.4% (17.7-23.0)	15.2% (13.5-16.9)	9.4% (7.3-11.6)
Anglo-Saxon	29.8% (25.8-33.8)	22.5% (18.5-26.6)	15.8% (12.2-19.4)
Southern	10.0% (8.0-11.9)	6.5% (4.0-8.9)	5.8% (2.9-8.6)
Eastern	23.7% (20.7-26.6)	20.6% (19.3-22.0)	6.4% (4.2-8.6)

For peer review only

For peer review only - http://bmjopen.bmj.com/site/about/guidelines.xhtml

Table 3 | Relative differences in paid employment between persons with and without a chronic illness by educational level in European regions in 2014

Region in Europe	Relative difference (PR) (95% CI)		
	Within low education	Within intermediate education	Within high education
**Male**			
Nordic	1.61 (1.46-1.77)	1.25 (1.21-1.30)	1.13 (1.10-1.17)
Continental	1.49 (1.40-1.58)	1.24 (1.21-1.27)	1.09 (1.07-1.12)
Anglo-Saxon	1.79 (1.64-1.95)	1.25 (1.17-1.33)	1.17 (1.12-1.23)
Southern	1.43 (1.38-1.49)	1.16 (1.12-1.20)	1.06 (1.03-1.09)
Eastern	1.94 (1.78-2.11)	1.42 (1.39-1.45)	1.11 (1.08-1.15)
**Female**			
Nordic	1.65 (1.48-1.84)	1.30 (1.24-1.36)	1.12 (1.09-1.15)
Continental	1.48 (1.39-1.58)	1.24 (1.20-1.27)	1.12 (1.09-1.15)
Anglo-Saxon	1.89 (1.70-2.11)	1.44 (1.33-1.57)	1.23 (1.17-1.30)
Southern	1.28 (1.21-1.34)	1.11 (1.07-1.16)	1.08 (1.04-1.12)
Eastern	1.76 (1.62-1.92)	1.37 (1.33-1.40)	1.08 (1.05-1.10)

For peer review only

For peer review only - http://bmjopen.bmj.com/site/about/guidelines.xhtml

Table 4 | Trends in absolute health-related inequalities in paid employment in European regions over the period 2005-2014

Region in Europe	Trend*		
	Low education	Intermediate education	High education
**Male**			
Nordic	0.009 (0.004-0.014)	0.001 (-0.002-0.003)	0.001 (-0.003-0.005)
Continental	0.003 (-0.001-0.007)	0.001 (0.000-0.003)	-0.002 (-0.005-0.000)
Anglo-Saxon	-0.003 (-0.013-0.007)	0.005 (-0.002-0.012)	0.003 (-0.002-0.007)
Southern	**-0.005 (-0.009--0.002)**	**-0.003 (-0.006-0.001)**	**-0.006 (-0.009--0.001)**
Eastern	0.002 (-0.004-0.009)	-0.001 (-0.004-0.002)	0.000 (-0.003-0.003)
**Female**			
Nordic	0.006 (-0.003-0.015)	-0.001 (-0.005-0.003)	-0.002 (-0.007-0.002)
Continental	**0.008 (0.004-0.011)**	**0.004 (0.003-0.006)**	0.002 (-0.002-0.005)
Anglo-Saxon	**0.006 (0.000-0.013)**	**0.008 (0.002-0.014)**	0.002 (-0.005-0.008)
Southern	-0.001 (-0.004-0.002)	0.001 (-0.003-0.005)	-0.003 (-0.008-0.003)
Eastern	**0.004 (0.000-0.008)**	0.002 (-0.001-0.004)	**-0.003 (-0.005-0.000)**

* Trend describes the widening (positive value) or narrowing (negative value) of the absolute difference of proportion in paid employment between participants with and without a chronic illness

For peer review only

Table 5 | Trends in relative health-related inequalities in paid employment in European regions over the period 2005-2014

Region in Europe	Trend*		
	Low education	Intermediate education	High education
**Male**			
Nordic	**0.026 (0.012-0.040)**	0.002 (-0.003-0.007)	0.001 (-0.004-0.006)
Continental	**0.010 (0.001-0.020)**	0.002 (-0.001-0.005)	**-0.003 (-0.007-0.000)**
Anglo-Saxon	0.014 (-0.024-0.053)	0.014 (-0.001-0.029)	0.005 (-0.002-0.012)
Southern	-0.001 (-0.007-0.004)	-0.002 (-0.007-0.002)	**-0.006 (-0.011--0.001)**
Eastern	0.013 (-0.008-0.035)	-0.004 (-0.010-0.002)	0.000 (-0.004-0.004)
**Female**			
Nordic	0.028 (-0.004-0.060)	-0.001 (-0.008-0.006)	-0.003 (-0.009-0.002)
Continental	**0.015 (0.002-0.028)**	**0.004 (0.001-0.007)**	0.001 (-0.004-0.007)
Anglo-Saxon	0.037 (-0.003-0.077)	**0.020 (0.006-0.034)**	0.003 (-0.007-0.013)
Southern	-0.007 (-0.016-0.001)	0.003 (-0.004-0.011)	-0.003 (-0.010-0.005)
Eastern	0.010 (-0.011-0.031)	0.000 (-0.007-0.006)	-0.002 (-0.005-0.002)

* Trend describes the widening (positive value) or narrowing (negative value) of the relative difference of prevalence ratios in paid employment between participants with and without a chronic illness

For peer review only

Acknowledgement: We kindly acknowledge the help of the Microdata Access team from Eurostat for provision of the EU-SILC datasets to our research entity (2014/044/NL).

Contributorship: JLDS prepared the data, conducted the analysis, and drafted and revised the paper. MS and KMOH participated in the analysis and commented on the paper. AB conceptualised the study, participated in the analysis and commented on the paper. He is the guarantor. All authors approved the final version. We kindly thank the WORKLONG Impact Group, in particular Lars Andersen, professor at the Danish National Research Centre for the Working Environment, and Manuel Flores, economist at the Directorate for Employment, Labour and Social Affairs at the Organisation for Economic Co-operation and Development, for their comments on the draft of the paper.

Funding: This work was conceived with financial support from award no. 208060001 by ZonMW within the Joint Programming Initiative More Years Better Lives (WORKLONG project) framework. Additional financial support was received through EIT Health. The sponsor had no role in the study design; in the collection, analysis, and interpretation of data; in the writing of the report; and in the decision to submit the article for publication.

Competing interests: All authors have completed the ICMJE uniform disclosure form at www.icmje.org/coi_disclosure.pdf and declare: financial support from the Joint Programming Initiative More Years Better Lives for the submitted work: no financial relationships with any organisations that might have an interest in the submitted work in the previous three years; no other relationships or activities that could appear to have influenced the submitted work.

Ethical approval: Not required.

Data sharing: No additional data available.

The lead author (AB) affirms that the manuscript is an honest, accurate, and transparent account of the study being reported; that no important aspects of the study have been omitted; and that any discrepancies from the study as planned (and, if relevant, registered) have been explained.

This is an Open Access article distributed in accordance with the Creative Commons Attribution Non Commercial (CC BY-NC 4.0) license, which permits others to distribute, remix, adapt, build upon this work non-commercially, and license their derivative works on different terms, provided the original work is properly cited and the use is noncommercial. See: https://creativecommons.org/licenses/by-nc/4.0/.

For peer review only

Figure 1 – Legend

High, not chronic	Intermediate, not chronic	Low, not chronic
High, chronic	Intermediate, chronic	Low, chronic

Figure 2 - Legend

Relative educational inequality in paid employment among persons with a chronic illness

For peer review only

For peer review only - http://bmjopen.bmj.com/site/about/guidelines.xhtml

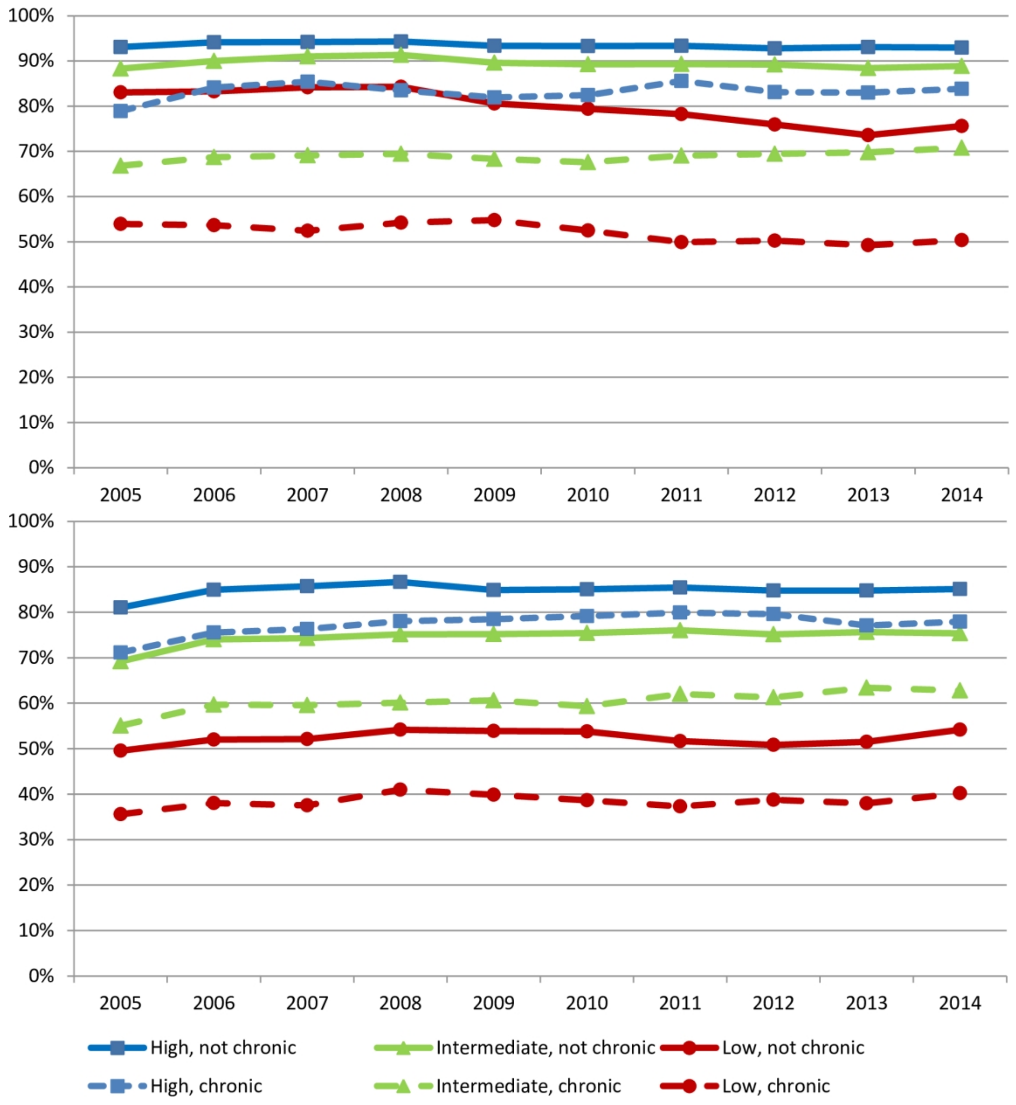

Figure 1 Proportion in paid employment with and without chronic illness by educational level – men (above) and women (below) in 26 European countries (2005-2014)

167x182mm (300 x 300 DPI)

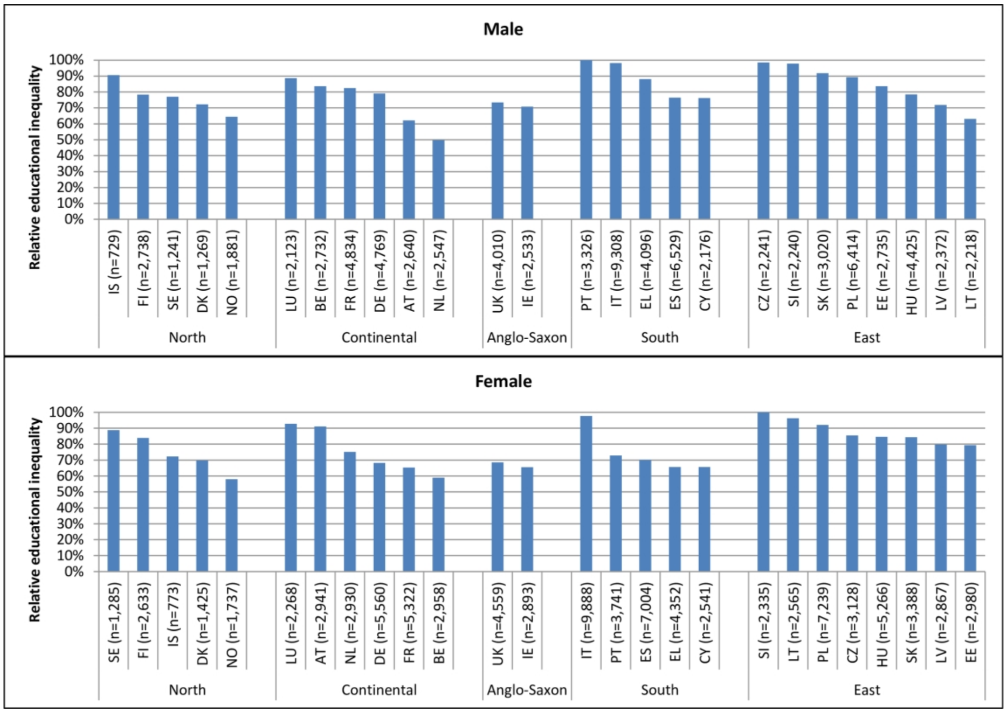

Figure 2 Relative inequality in paid employment among persons with a chronic illness comparing low educated persons with high educated persons

190x134mm (300 x 300 DPI)

Supplementary table 1 | Absolute differences in paid employment between persons with and without a chronic illness by educational level in European regions between 2005-2014 - male

Region in Europe	Absolute difference (%)									
	2005	2006	2007	2008	2009	2010	2011	2012	2013	2014
**Low education**										
Nordic	26.1	27.0	25.2	25.7	26.7	32.9	29.6	32.6	31.4	32.9
Continental	22.9	23.0	24.5	25.9	24.3	24.6	29.2	24.7	25.2	26.1
Anglo-Saxon	38.7	35.6	37.2	40.4	27.3	32.2	38.9	34.6	34.7	35.3
Southern	23.7	24.5	25.6	26.1	23.5	22.1	22.1	21.7	19.6	21.5
Eastern	28.6	31.4	36.9	35.8	30.9	32.5	32.7	35.5	32.8	33.5
**Intermediate education**										
Nordic	19.2	18.5	18.4	16.8	18.8	20.8	19.0	18.9	19.1	18.6
Continental	16.4	15.9	15.5	16.4	18.2	17.2	16.6	17.3	17.0	17.2
Anglo-Saxon	17.9	17.7	18.5	18.5	18.0	23.9	20.3	23.7	25.7	17.1
Southern	12.4	12.4	12.7	13.3	14.9	13.7	11.9	11.2	9.6	11.2
Eastern	26.0	26.6	27.5	28.7	27.1	27.8	26.9	27.5	25.3	25.5
**High education**										
Nordic	10.7	8.2	8.6	8.0	9.3	7.3	6.6	9.3	10.7	11.1
Continental	11.4	9.5	8.8	8.3	7.7	8.3	7.8	7.7	8.7	8.2
Anglo-Saxon	12.1	11.5	10.6	14.6	14.4	16.4	11.9	15.8	13.4	13.4
Southern	9.3	10.5	6.6	8.9	10.9	7.8	6.1	6.9	5.5	5.0
Eastern	10.5	11.9	9.8	12.3	11.2	12.7	10.2	12.1	11.6	9.8

For peer review only

Supplementary table 2 | Absolute differences in paid employment between persons with and without a chronic illness by educational level in European regions between 2005-2014 - female

Region in Europe	Absolute difference (%)									
	2005	2006	2007	2008	2009	2010	2011	2012	2013	2014
**Low education**										
Nordic	31.6	34.1	33.3	31.0	32.9	34.0	34.9	42.0	41.1	31.0
Continental	15.9	15.3	13.6	14.7	16.3	19.0	18.4	19.0	21.9	20.4
Anglo-Saxon	25.7	20.5	27.9	25.6	23.3	23.6	25.7	29.3	28.9	29.8
Southern	8.6	10.1	10.7	11.1	9.1	9.6	10.8	8.0	7.7	10.0
Eastern	20.2	20.7	21.9	19.4	22.1	20.5	19.8	24.6	23.4	23.7
**Intermediate education**										
Nordic	18.9	18.2	21.8	20.7	18.9	17.4	19.4	20.3	16.7	20.1
Continental	10.7	11.3	12.0	12.7	13.3	13.9	12.7	14.0	13.7	15.2
Anglo-Saxon	14.5	13.9	14.2	18.0	17.4	14.7	13.0	18.9	21.8	22.5
Southern	5.2	4.8	9.1	5.7	5.5	6.8	5.2	5.6	8.8	6.5
Eastern	19.1	19.9	21.8	21.8	22.0	22.1	21.8	22.9	21.1	20.6
**High education**										
Nordic	8.1	11.8	11.4	9.1	9.6	9.0	6.9	6.2	9.2	9.6
Continental	8.2	8.7	7.9	9.0	6.2	7.4	8.8	7.2	11.7	9.4
Anglo-Saxon	13.7	11.5	13.4	11.9	9.3	9.0	8.1	13.0	14.4	15.8
Southern	4.5	9.4	6.2	2.5	2.8	3.1	3.6	2.6	3.5	5.8
Eastern	10.0	8.2	9.9	8.6	8.0	9.2	9.1	8.4	7.5	6.4

For peer review only

Supplementary table 3 | Relative differences in paid employment between persons with and without a chronic illness by educational level in European regions between 2005-2014 - male

Region in Europe	Relative difference (PR)									
	2005	2006	2007	2008	2009	2010	2011	2012	2013	2014
**Low education**										
Nordic	1.43	1.43	1.39	1.39	1.45	1.62	1.53	1.60	1.58	1.61
Continental	1.40	1.40	1.43	1.46	1.43	1.45	1.56	1.45	1.46	1.49
Anglo-Saxon	1.86	1.74	1.83	2.05	1.66	1.88	2.14	1.95	2.02	1.79
Southern	1.40	1.42	1.43	1.44	1.41	1.38	1.40	1.41	1.38	1.43
Eastern	1.80	1.89	2.05	1.93	1.85	1.96	1.99	2.11	1.95	1.94
**Intermediate education**										
Nordic	1.26	1.25	1.24	1.22	1.26	1.29	1.26	1.26	1.26	1.25
Continental	1.23	1.22	1.21	1.22	1.25	1.24	1.23	1.24	1.23	1.24
Anglo-Saxon	1.24	1.23	1.25	1.25	1.26	1.39	1.31	1.37	1.42	1.25
Southern	1.16	1.16	1.16	1.17	1.20	1.18	1.16	1.15	1.13	1.16
Eastern	1.46	1.46	1.46	1.48	1.47	1.50	1.47	1.48	1.42	1.42
**High education**										
Nordic	1.13	1.09	1.10	1.09	1.11	1.08	1.07	1.11	1.13	1.13
Continental	1.14	1.11	1.10	1.09	1.09	1.10	1.09	1.09	1.10	1.09
Anglo-Saxon	1.15	1.14	1.13	1.18	1.19	1.22	1.15	1.21	1.17	1.17
Southern	1.11	1.13	1.08	1.11	1.13	1.09	1.07	1.08	1.07	1.06
Eastern	1.12	1.14	1.11	1.15	1.13	1.16	1.12	1.15	1.14	1.11

For peer review only

Supplementary table 4 | Relative differences in paid employment between persons with and without a chronic illness by educational level in European regions between 2005-2014 - female

Region in Europe	Relative difference (PR)									
	2005	2006	2007	2008	2009	2010	2011	2012	2013	2014
**Low education**										
Nordic	1.63	1.67	1.64	1.58	1.66	1.72	1.74	2.02	1.96	1.65
Continental	1.44	1.38	1.32	1.33	1.37	1.45	1.44	1.45	1.53	1.48
Anglo-Saxon	1.84	1.59	2.02	1.86	1.79	1.86	2.04	2.16	2.22	1.89
Southern	1.27	1.31	1.32	1.31	1.25	1.26	1.29	1.21	1.21	1.28
Eastern	1.70	1.77	1.78	1.63	1.77	1.71	1.68	1.94	1.80	1.76
**Intermediate education**										
Nordic	1.28	1.27	1.32	1.30	1.27	1.25	1.28	1.30	1.24	1.30
Continental	1.19	1.18	1.20	1.21	1.21	1.22	1.19	1.22	1.21	1.24
Anglo-Saxon	1.25	1.23	1.23	1.30	1.30	1.26	1.22	1.34	1.41	1.44
Southern	1.08	1.08	1.16	1.09	1.09	1.11	1.09	1.10	1.16	1.11
Eastern	1.38	1.39	1.42	1.41	1.42	1.43	1.41	1.44	1.38	1.37
**High education**										
Nordic	1.10	1.15	1.14	1.11	1.12	1.11	1.08	1.07	1.11	1.12
Continental	1.11	1.12	1.10	1.12	1.08	1.09	1.11	1.09	1.15	1.12
Anglo-Saxon	1.20	1.16	1.19	1.16	1.13	1.13	1.11	1.19	1.21	1.23
Southern	1.05	1.12	1.07	1.02	1.03	1.04	1.04	1.03	1.04	1.08
Eastern	1.11	1.09	1.12	1.10	1.10	1.11	1.11	1.10	1.09	1.08

For peer review only

STROBE Statement—checklist of items that should be included in reports of observational studies

	Item No	Recommendation	Reported on Page No.
**Title and abstract**	1	(*a*) Indicate the study's design with a commonly used term in the title or the abstract	1, 3
		(*b*) Provide in the abstract an informative and balanced summary of what was done and what was found	3
**Introduction**			
Background/rationale	2	Explain the scientific background and rationale for the investigation being reported	4
Objectives	3	State specific objectives, including any prespecified hypotheses	4,5
**Methods**			
Study design	4	Present key elements of study design early in the paper	6-8
Setting	5	Describe the setting, locations, and relevant dates, including periods of recruitment, exposure, follow-up, and data collection	6
Participants	6	(*a*) *Cohort study*—Give the eligibility criteria, and the sources and methods of selection of participants. Describe methods of follow-up	n.a.
		Case-control study—Give the eligibility criteria, and the sources and methods of case ascertainment and control selection. Give the rationale for the choice of cases and controls	n.a.
		Cross-sectional study—Give the eligibility criteria, and the sources and methods of selection of participants	6
		(*b*) *Cohort study*—For matched studies, give matching criteria and number of exposed and unexposed	n.a.
		Case-control study—For matched studies, give matching criteria and the number of controls per case	n.a.
Variables	7	Clearly define all outcomes, exposures, predictors, potential confounders, and effect modifiers. Give diagnostic criteria, if applicable	6
Data sources/ measurement	8*	For each variable of interest, give sources of data and details of methods of assessment (measurement). Describe comparability of assessment methods if there is more than one group	6
Bias	9	Describe any efforts to address potential sources of bias	6
Study size	10	Explain how the study size was arrived at	6
Quantitative variables	11	Explain how quantitative variables were handled in the analyses. If applicable, describe which groupings were chosen and why	6
Statistical methods	12	(*a*) Describe all statistical methods, including those used to control for confounding	7-8
		(*b*) Describe any methods used to examine subgroups and interactions	7-8
		(*c*) Explain how missing data were addressed	6
		(*d*) *Cohort study*—If applicable, explain how loss to follow-up was addressed	n.a.
		Case-control study—If applicable, explain how matching of cases and controls was addressed	n.a.
		Cross-sectional study—If applicable, describe analytical methods	n.a.

For peer review only

taking account of sampling strategy	
(*e*) Describe any sensitivity analyses	n.a.

Continued on next page

For peer review only

Results			Reported on Page No.
Participants	13*	(a) Report numbers of individuals at each stage of study—eg numbers potentially eligible, examined for eligibility, confirmed eligible, included in the study, completing follow-up, and analysed	9, 17
		(b) Give reasons for non-participation at each stage	n.a.
		(c) Consider use of a flow diagram	n.a.
Descriptive data	14*	(a) Give characteristics of study participants (eg demographic, clinical, social) and information on exposures and potential confounders	9, 17
		(b) Indicate number of participants with missing data for each variable of interest	n.a.
		(c) *Cohort study*—Summarise follow-up time (eg, average and total amount)	n.a
Outcome data	15*	*Cohort study*—Report numbers of outcome events or summary measures over time	n.a.
		Case-control study—Report numbers in each exposure category, or summary measures of exposure	*n.a.*
		Cross-sectional study—Report numbers of outcome events or summary measures	9,17
Main results	16	(*a*) Give unadjusted estimates and, if applicable, confounder-adjusted estimates and their precision (eg, 95% confidence interval). Make clear which confounders were adjusted for and why they were included	9, 18-21
		(*b*) Report category boundaries when continuous variables were categorized	9, 18-21
		(*c*) If relevant, consider translating estimates of relative risk into absolute risk for a meaningful time period	n.a.
Other analyses	17	Report other analyses done—eg analyses of subgroups and interactions, and sensitivity analyses	9,10,25
**Discussion**			
Key results	18	Summarise key results with reference to study objectives	11-12
Limitations	19	Discuss limitations of the study, taking into account sources of potential bias or imprecision. Discuss both direction and magnitude of any potential bias	12
Interpretation	20	Give a cautious overall interpretation of results considering objectives, limitations, multiplicity of analyses, results from similar studies, and other relevant evidence	11-12
Generalisability	21	Discuss the generalisability (external validity) of the study results	12
**Other information**			
Funding	22	Give the source of funding and the role of the funders for the present study and, if applicable, for the original study on which the present article is based	22

*Give information separately for cases and controls in case-control studies and, if applicable, for exposed and unexposed groups in cohort and cross-sectional studies.

Note: An Explanation and Elaboration article discusses each checklist item and gives methodological background and published examples of transparent reporting. The STROBE checklist is best used in conjunction with this article (freely available on the Web sites of PLoS Medicine at http://www.plosmedicine.org/, Annals of Internal Medicine at http://www.annals.org/, and Epidemiology at http://www.epidem.com/). Information on the STROBE Initiative is available at www.strobe-statement.org.

For peer review only

For peer review only - http://bmjopen.bmj.com/site/about/guidelines.xhtml

BMJ Open

Health-related educational inequalities in paid employment across 26 European countries in 2005-2014: a repeated cross-sectional study

Journal:	*BMJ Open*
Manuscript ID	bmjopen-2018-024823.R2
Article Type:	Research
Date Submitted by the Author:	30-Dec-2018
Complete List of Authors:	Schram, Jolinda; Erasmus MC, Department of Public Health<br>Schuring, Merel; Erasmus MC<br>Oude Hengel, Karen; Erasmus MC; Netherlands Organization for Applied Scrientific Research TNO<br>Burdorf, Alex; Erasmus MC, Department of Public Health
**Primary Subject Heading**:	Public health
Secondary Subject Heading:	Global health
Keywords:	Trend analysis, Social inequalities, Chronic illness, Employment

SCHOLARONE™
Manuscripts

Title **Health-related educational inequalities in paid employment across 26 European countries in 2005-2014: a repeated cross-sectional study**

Authors' names Jolinda LD Schram[1], Merel Schuring[1], Karen M Oude Hengel[1,2], Alex Burdorf[1]

Corresponding author Correspondence to: Merel Schuring, Erasmus University Medical Center, Department of Public Health, P.O. Box 2040, 3000 CA Rotterdam, The Netherlands, m.schuring@erasmusmc.nl , +31107038993

Affiliation [1] Erasmus University Medical Center, Department of Public Health, P.O. Box 2040, 3000 CA Rotterdam, The Netherlands.
[2] Netherlands Organization for Applied Scientific Research TNO, P.O. Box 3005, 2301 DA Leiden, The Netherlands.

Key words Social inequalities, Employment, Chronic illness, Trend analysis
Word count 3,943

For peer review only

For peer review only - http://bmjopen.bmj.com/site/about/guidelines.xhtml

ABSTRACT

Objective: The study investigates trends in health-related inequalities in paid employment among men and women in different educational groups in 26 countries in five European regions.

Design: Individual-level analysis of repeated cross-sectional annual data (2005-2014) from the EU Statistics on Income and Living Conditions (EU-SILC).

Setting: 26 European countries in five European regions.

Participants: 1,844,915 individuals age 30-59 years were selected with information on work status, chronic illness, educational background, age, and gender.

Outcome measures: Absolute differences were expressed by absolute differences in proportion in paid employment between participants with and without a chronic illness, using linear regression. Relative differences were expressed by prevalence ratios in paid employment, using a Cox proportional hazard model. Linear regression was used to examine the trends of inequalities.

Results: Participants with a chronic illness had consistently lower labour force participation than those without illnesses. Educational inequalities were substantial with absolute differences larger within lower educated (men 21-35%, women 10-31%) than within higher educated (men 5-13%, women 6-16%). Relative differences showed that low educated men with a chronic illness were 1.4-1.9 times (women 1.3-1.8 times) more likely to be out of paid employment than low educated persons without a chronic illness, whereas this was 1.1-1.2 among high educated men and women. In the Nordic, Anglo-Saxon and Eastern regions these health-related educational inequalities in paid employment were more pronounced than in the Continental and Southern region. For most regions, absolute health-related educational inequalities in paid employment were generally constant, whereas relative inequalities increased, especially among low educated persons.

Conclusions: Men and women with a chronic illness have considerable less access to the labour market than their healthy colleagues, especially among lower educated persons. This exclusion from paid employment will increase health inequalities.

For peer review only

Strengths and limitations of the study

- This is the first study to present information on trends in health-related educational inequalities in paid employment among persons in different European regions
- A strength of the current study is the use of cross sectional data from a large number of European countries and large number of individuals for which the outcomes are harmonized (1,844,915 individuals in 26 countries).
- The effects of having a chronic illness on being employed (selection) and unemployment causing ill health such as chronic illness (causation), cannot be distinguished by using cross-sectional data.
- Although the outcomes of EU-SILC are harmonized, comparisons in self-rated health measures between different cultures need to be made with caution.

For peer review only

For peer review only - http://bmjopen.bmj.com/site/about/guidelines.xhtml

Introduction

Life expectancy continues to increase and will ensure that the old-age dependency ratio, the number of elderly people as a share of those of working age, will rise steadily from 29.4 in 2016 to 50.3 by 2050 in Europe (1, 2). In response, many countries have raised the statutory retirement age (3). The challenge is ensuring that those of working age are actually participating in the labour force (4). Health-related exclusion from the labour market is present in many countries (5, 6), whereby workers with a chronic illness are at increased risk of becoming disabled or unemployed. Leaving paid employment may further impact health negatively (7).

Studies have shown that the presence of a chronic disease is an important barrier for entering and maintaining paid employment (5). Educational inequalities in morbidity, such as chronic health conditions, are well documented across European countries (8). Some studies have suggested that the effect of chronic illness on maintaining paid employment differs by socioeconomic position. Bartley and Owen reported that workers with a chronic disease in lower socioeconomic position had more difficulties staying in paid employment than those with a higher socioeconomic position during times of economic crisis (9). In Sweden and the United Kingdom labour market attachment for those with a chronic illness is reduced, with the effect being substantially stronger in the United Kingdom than in Sweden (10, 11).

The differential effect of socioeconomic position on the association between chronic illness and paid employment may be modified by national social and economic conditions, such as labour market policies (12). European countries can be categorized in five regions: the Nordic, Continental, Anglo-Saxon, Southern, and Eastern regions (13, 14). This typology reflects general differences in employment protection and income security. Non-employment rates for persons with a longstanding illness as well as a low educational level were found particularly high in the Anglo-Saxon and Eastern regions, compared to the Nordic region (15). The poor position of disabled people was suggested to be partly counteracted by employment protection (16), although inequalities in health in itself were not consistently smaller in the Nordic region (17).

Few studies have documented the trends over time in socioeconomic differences in labour force participation between persons with and without a chronic illness. A repeated cross sectional study over 25 years in Britain showed that within the lower occupational class persons with a limiting illness had greater and more rapidly widening gaps in labour force participation than in the higher occupational class (18). A comparison between the Nordic countries and the United Kingdom showed that, since the mid-1990s, there has been a deterioration in paid employment among persons with limiting long standing illness with a low education, even when the economy was doing well (19).

Limited information exists on trends in health-related educational inequalities in paid employment among persons in different European regions, since most studies have focused on a single country or compared a selected group of countries. Therefore, the aims of this paper are (i) to examine absolute and relative differences in paid employment between persons with and without a chronic illness within each educational group in five European regions and (ii) to describe trends in socioeconomic differences in paid employment between people with and without a chronic illness within European countries.

For peer review only - http://bmjopen.bmj.com/site/about/guidelines.xhtml

Methods

Data

A repeated cross-sectional study was conducted with the EU-SILC survey (European Union Statistics on Income and Living Conditions). The EU-SILC is a survey questionnaire designed to provide comparable information across the EU and cross-sectional data from 2005 to 2014 was used from 26 countries that participated in EU-SILC since 2005 (Denmark, Finland, Iceland, Norway, Sweden, Austria, Belgium, Germany, France, Luxembourg, The Netherlands, Cyprus, Greece, Italy, Portugal, Spain, Ireland, United Kingdom, Czech Republic, Estonia, Hungary, Latvia, Lithuania, Poland, Slovakia and Slovenia). Detailed information on the EU-SILC study design can be found elsewhere (20).

Respondents were selected with information on work status, chronic illness, educational background, age, and gender. After excluding individuals who were younger than 30 or older than 59 (around 50% of the sample), and deleting observations with relevant missing information (almost all due to no information on chronic disease, 16% of the remaining sample), our study comprised 179,724 individuals for 2014. For the trend analysis 1,844,915 individuals were selected aged 30-59 years between 2005-2014. The age limit of 59 years was applied because a number of countries have an official retirement age of 60, thereby greatly reducing the proportion of employed persons after 60 years. The age limit of 30 years was used to exclude potential confounding due to different educational pathways between those with a chronic illness and those without.

Employment status

Employment status was classified into six mutually exclusive categories: Employed (working full or part-time, self-employed full or part-time), unemployed, economically inactive (fulfilling domestic tasks and care responsibilities, and other inactivity), retired, disabled and other (student, in compulsory military community or service). For the main analysis a dichotomized variable was used: paid employment or outside of paid employment (all other options).

Chronic illness

The question *Do you have any longstanding illness or health problem?* was used to distinguish between people with or without a chronic illness.

Sociodemographic variables

Three sociodemographic variables were used in the study: age, gender, and education. Age was categorized into three 10-year age groups: 30-39, 40-49 and 50-59 years. Educational level was recorded as the highest level of education completed by the respondent. We coded education according to the 1997 International Standard Classification of Education (ISCED-97) and categorized as low (0-2: pre-primary, primary and lower secondary education), intermediate (3-4: upper secondary education/post-secondary non tertiary) and high (5-6: tertiary education).

Regions

The European countries were categorized into five different regions, based on relative comparability of welfare state regimes and geography (13). The Nordic region included Denmark, Finland, Iceland, Norway, and Sweden. The Continental region included Austria, Belgium, Germany, France, Luxembourg, and The Netherlands. The Southern region included Cyprus, Greece, Italy, Portugal, and Spain. The Anglo-Saxon countries included Ireland and the United Kingdom. The Eastern region included the Czech Republic, Estonia, Hungary, Latvia, Lithuania, Poland, Slovakia, and Slovenia.

For peer review only - http://bmjopen.bmj.com/site/about/guidelines.xhtml

Statistical analysis

The statistical analysis focuses on both absolute and relative differences in labour force participation among those with and without a chronic disease across different educational groups. The absolute difference depicts the reduction of being in paid employment among persons with a chronic illness and is of special interest as it presents the impact on the total population in %point employment rate. The relative difference presents the prevalence ratio as a measure of the strength of the association between having a chronic illness and being out of paid employment. Absolute and relative inequalities may differ because levels of labour force participation are generally higher in higher educated groups.

Annual sample weights were used to estimate the prevalence of labour market participation that are representative for the countries included in this study. For each region the association of having a chronic illness with labour force participation within each educational group was analysed using two different models. A linear regression model was used to calculate absolute differences in labour force participation between persons with and without a chronic illness. Cox regression analysis with a robust estimate was applied to calculate relative differences.

The linear regression model to calculate absolute difference can be written as:

$$y = \alpha + \beta_1 low + \beta_2 intermediate + \beta_3 low\_chronic + \beta_4 intermediate\_chronic + \beta_5 high\_chronic + \beta_6 age$$

where *y* is a dichotomous variable for being employed; *α* is an intercept, two dummy variables to compare low and intermediate education with high education, and three additional dummy variables to compare those with a chronic illness to those without in each education group. Adjustment for age was done by default. In this binary linear regression analysis the dependent variable expresses the proportional difference of being in paid employment (within the range of 0.0 to 1.0), comparing subjects with a chronic disease to subjects without a chronic disease in the same educational group. Thus, the proportional difference equals the absolute difference or gap between proportions of being in paid employment, which is our primary measure of interest (21).

The Cox proportional hazard model to calculate relative differences can be written as:

$$h(t,x) = h_0(t)\exp\left(\sum_{k=1}^{p} \beta_1 low + \beta_2 intermediate + \beta_3 low\_chronic + \beta_4 intermediate\_chronic + \beta_5 high\_chronic + \beta_6 age\right)$$

where *h(t,…)* is the likelihood at time *t* that a person is not employed, two dummy variables for low and intermediate education to compare to high education, and three dummy variables to compare those with a chronic illness in each education group. Adjustment for age was done by default. Cox regression analysis was preferred above a logistic regression analysis, since it will more accurately estimate the prevalence ratio , which is much closer to the relative risk between being in paid employment and having a chronic illness, than the odds ratios in a logistics regression analysis, since the odds ratio will overestimate the risk ratio due to the high prevalence of being in paid employment in the current study (22, 23).

The time period for all persons was set at a constant level (value 1) to estimate the prevalence ratio (PR) (22, 24). In all analyses, variables were recoded so a PR above 1 indicates an increased likelihood of being out of paid employment with corresponding 95% confidence intervals (95% CI). In order to

For peer review only - http://bmjopen.bmj.com/site/about/guidelines.xhtml

account for differences in labour market participation between men and women across regions (25), the associations were stratified by sex and region , and adjusted for age. Significance was determined by P < 0.05.

Trend analysis in the five regions

For determining the trends in absolute and relative health-related inequalities in being in paid employment over 10 years in each region, we used linear regression analyses with the absolute gap and prevalence ratios in each educational group as dependent variable in each survey year. The analysis was stratified by region and gender. The year of data collection was used as the independent variable.

Health-related educational inequalities in paid employment

In order to estimate educational inequalities in the relative differences in labour force participation among persons with a chronic illness a counterfactual scenario was introduced. In this scenario it is assumed that the relative difference in labour force participation between those with a chronic illness and those without in the lower educational group can be reduced to the relative difference observed in the higher educational group (26). The estimation of the excess relative difference in paid employment for chronic illness status in lower educated workers is a fair reflection of educational inequalities between low and high educated persons. The Cox proportional hazard model was used to estimate this excess relative difference by the formula: (bèta for relative difference in the low educated group – bèta for relative difference in the high educated group)/(bèta of the relative difference in the low educated group).

The main analysis was performed using Stata statistical software version 14 (Stata-Corp, College Station, Texas). The estimates of paid employment per country and the trend analysis were conducted in IBM SPSS statistical software version 22 (SPSS Inc, Chicago, IL, USA).

Patient involvement

Not applicable, we have used available datasets from Eurostat for secondary data analysis.

For peer review only - http://bmjopen.bmj.com/site/about/guidelines.xhtml

Results

The study population consisted of 179,724 participants of whom half were women, the largest group was intermediately educated, 75% were employed, and 27% had a chronic illness. The Eastern region had a large intermediate educated group (64%), whereas the Southern and Anglo-Saxon regions had a relatively large low educated group (28 and 38%, respectively) (Table 1). The Southern region had the lowest labour force participation, and the lowest percentage of people with a chronic illness. The Nordic region had the highest employment, but also the highest prevalence of people with a chronic illness.

Labour force participation over the period 2005-2014 among men without a chronic illness remained stable for intermediate and high educated persons, but decreased by about 5% among those with lower education (Figure 1A). Within each educational group, labour force participation among men with a chronic illness was substantially lower than among men without illness, with the largest difference among the lowest educated persons. For women the labour force participation increased slightly for most groups over the period 2005-2014 (Figure 1B). Women had a consistently lower labour force participation than men with increasing differences by lower educational level. Women with a chronic disease had substantially lower labour force participation than women without a chronic illness.

The absolute differences in labour force participation between persons with and without a chronic illness increased with lower educational level, among men and women (Table 2). The absolute differences were larger for men than for women. For low educated persons the absolute differences in labour force participation between men with and without a chronic illness were higher in the Nordic, Anglo-Saxon and Eastern region (32.9-35.3%) than in the Continental and Southern region (21.5-26.1%). Among women a comparable pattern was observed.

A similar pattern was found for relative differences in health-related labour force participation (Table 3). Overall, low educated men with a chronic illness were 1.4 to 1.9 times more likely to be outside of paid employment than low educated men without a chronic illness, whereas this was 1.1 to 1.2 times within the high educational group. Relative differences in labour force participation among low educated men without and with a chronic illness were the highest in the Anglo-Saxon and Eastern region (1.79-1.94). For men with high education modest regional differences were found in labour force participation between those without and with a chronic illness. Comparable patterns were observed for women.

Trends in absolute differences in labour force participation showed stable inequalities for men in all educational groups in most regions (Table 4, Suppl. Table 1,2). Among men, significant narrowing of absolute health-related inequalities in paid employment was observed for all educational categories in the Southern region over the period 2005-2014. For women, significant widening of absolute health-related inequalities in paid employment were observed for low and intermediate educated women in the Continental and Anglo-Saxon region. In the Eastern region significant widening in inequalities was observed for low educated women, while narrowing inequalities were observed for high educated women.

Trends in relative differences in labour force participation were constant among high educated men and widened in low educated men (Table 5, Suppl. Table 3,4). Among men, significant widening of relative differences were observed for lower educated persons in the Nordic and Continental region of respectively 1% and 2.6% over the period 2005-2014. Significant narrowing of relative health-related inequalities in paid employment was observed among higher educated men in the Continental and Southern region (-0.3% and -0.6%). Trends for women were similar. In most regions relative health-related inequalities in paid employment were widening for low educated women

For peer review only - http://bmjopen.bmj.com/site/about/guidelines.xhtml

(between 1.0% and 3.7%), although in the Southern region there was a small narrowing (-0.7%). For high educated women there was no clear trend.

Figure 2 presents the inequalities between low and high educated persons for the influence of a chronic illness on being in paid employment within each educational group. The excess relative difference among low educated persons across 26 European countries varied between 50% to 100% among men and between 58% and 100% among women when compared to high educated persons. The variation in educational inequalities in labour market position of those with a chronic disease was larger within European regions than between European regions, indicating substantial differences between countries with supposedly similar welfare regimes.

For peer review only

For peer review only - http://bmjopen.bmj.com/site/about/guidelines.xhtml

Discussion

Within Europe, persons with a chronic disease were less often in paid employment than persons without a chronic disease in the period 2005-2014. The low education group had the largest absolute and relative differences in paid employment between persons with and without a chronic illness. For men these differences were more pronounced than for women. The Nordic, Anglo-Saxon and Eastern regions had substantially higher relative and absolute differences in paid employment than in the Continental and Southern region. The relative position on the labour market for persons with a chronic illness worsened in the past 10 years among low educated persons, and remained fairly stable for high educated persons.

The risk of being outside of paid employment with a chronic illness is higher for persons with a low education than those with a high education. Previous studies have shown comparable results in specific countries (9, 11, 16, 18) or across Europe in one particular year. (27, 28) Our study expands this knowledge by demonstrating that these patterns are consistent over time and European countries, and by illustrating that health-related educational inequalities in paid employment have widened rather than narrowed in the past decade.

Men and women with a chronic illness had less access to the labour market than their healthy colleagues, especially among lower educated persons. Possible explanations for the observed educational inequality relate to severity of chronic illnesses and quality of jobs. In this study chronic illness could not be differentiated by diagnosis and severity. It may be hypothesised that lower educated workers more often suffer from chronic diseases with functional limitations than high educated workers (29, 30). Having a chronic illness and functional limitations follows a social gradient in our data (high educated 49%, low educated 73%). The education inequality may also be explained by differences in strenuous work and other job characteristics that may interfere with health problems (31, 32).

A few studies have demonstrated that strenuous work conditions hamper workers with a chronic illness to remain in paid employment. Among Finnish workers physical work load strongly mediated the association between musculoskeletal disorders and exit from paid employment (33). A Dutch study attributed 12% of educational differences in disability benefits to work characteristics (34). Another Dutch study showed that high autonomy, high social support and low psychosocial job strongly moderated the association between chronic health conditions and exit through disability benefits (35). Lower socio-economic jobs more often have unfavourable work characteristics (36). Therefore, the ability to remain in paid employment is reduced for low educated persons with a chronic illness.

Health-related absolute and relative differences in paid employment across educational groups were consistently lower in the Southern region than in other European regions. A few studies (12, 18, 27) have noted larger absolute and relative differences in labour force participation between persons with and without a chronic illness in regions with low employment protection compared to regions with high employment protection. Our results in the Southern region, which is characterised by high employment protection, corroborates these previous studies. However, the educational inequalities in paid employment depicted in figure 2 showed a larger variation between countries in the same region than between different regions. This suggests that other social, legal and economic conditions, such as availability of social benefits, also play a role in being in paid employment of people with a chronic illness. More studies are necessary to evaluate the influence of various national indicators of social, cultural and economic conditions on the variation in health-related educational inequalities in paid employment.

For peer review only - http://bmjopen.bmj.com/site/about/guidelines.xhtml

The absolute and relative disadvantages in labour force participation among those with a chronic disease were more pronounced for men than for women in most welfare states. In the Southern welfare state regime the absolute and relative differences were smaller for women, but in the Nordic welfare state regime the two groups showed similar patterns. These regional differences may partly be explained by institutional support i.e. the full-employment policies of the Nordic region, facilitating the integration of women into the labour force by providing for example child care, although often the decision to be active or not is strongly affected by education and fertility choices (37, 38). The high labour force participation of women in Nordic welfare states can contribute to higher relative differences between those with and without a chronic illness compared to the Southern region. This is in line with previous studies stating that in terms of socioeconomic inequalities the relative inequalities do not appear to be smallest in more redistributive countries such as the Scandinavian welfare state, but that the absolute health of all social classes is often better (39).

The trends observed in this study showed that over the period 2005 to 2014 educational inequalities in paid employment of persons with a chronic illness have widened among low educated persons. In the recent decades precarious employment, such as temporary contracts and part-time work, have become more common in Europe (40, 41). Further research is necessary to evaluate whether the increase in precarious employment could be an explanation for increasing educational inequalities in paid employment.

The trend analysis might be influenced by the differential effect of the economic crisis across regions. The Continental and Eastern region have had an increase in persons in paid employment over the period 2005-2014, while the Southern region had a decrease of persons in paid employment due to the economic crisis. In periods of high and rapid increase in unemployment rates, workers in good health will also lose their jobs and, thus, the influence of ill health on paid employment will attenuate (42). This mechanism may explain why we found a decreasing trend in the Southern region. Furthermore, health may have worsened during the economic crisis. For persons with existing health issues, this could have led to more difficulties maintaining paid employment. A recent study found an increase in absolute inequalities in health between educational groups after the recent economic crisis in the Continental and Anglo-Saxon region (43). This may explain the increasing trends we found in these regions.

Strengths and limitations

Data were derived from the European Union Statistics on Income and Living Conditions (EU-SILC). A strength of the current study is the use of repeated cross sectional data from a large number of European countries for which the outcomes are harmonized. By utilizing Cox regression instead of logistic regression, differences in labour force attachment in EU SILC across countries will not affect the results of the statistical analysis. The repeated cross-sectional surveys present a snapshot of the labour force situation of each region for the respective years, but causal relations cannot be investigated in depth, as other studies with cohort information have done (44, 45). By using cross sectional data in this study the effects of having a chronic illness on paid employment (selection) and unemployment causing ill health such as chronic illness (causation), cannot be distinguished.

A limitation of using EU-SILC data is the variation in mode of data collection, translations, and cultural interpretation (27). Further limitations are the use of self-reported health and labour market measures which could vary by country, socioeconomic position and cultural beliefs (13). However, a number of studies have shown that self-rated measurements of health are strongly predictive for objective health measures, such as mortality (46, 47). Comparisons in self-rated health measures between different cultures do need to be made with caution (48).

For peer review only - http://bmjopen.bmj.com/site/about/guidelines.xhtml

Sixteen percent of our sample did not respond to the question on chronic illness. These non-respondents had a similar age, were higher educated and more often employed than respondents. Based on their characteristics, non-respondents were less likely to have a chronic illness. Although it is difficult to predict the influence of this selective reporting, the slight underrepresentation of subjects with a chronic disease may have resulted in an underestimation of the association between having a chronic illness and being in paid employment.

Policy implications

Men and women with a chronic illness have considerable less access to the labour market than their healthy colleagues, especially among lower educated persons. Their position in the labour market seems to have worsened rather than improved in the past decade. Persons with a chronic illness without a paid job are at risk for further deterioration of their health and economic marginalisation. More effective policy measures are needed to reduce the gap in paid employment between those with and without a chronic illness, especially in the low education group, in all European regions.

For peer review only

References

1. Eurostat. Projected old-age dependency ratio: Eurostat; 2013 [updated 11 September 2017. Available from: http://ec.europa.eu/eurostat/tgm/table.do?tab=table&init=1&language=en&pcode=tsdde511&plugin=1.
2. Eurostat. Old-age-dependency ratio: Eurostat; 2017 [updated 11 September 2017. Available from: http://ec.europa.eu/eurostat/tgm/table.do?tab=table&init=1&language=en&pcode=tsdde510&plugin=1.
3. Christensen K, Doblhammer G, Rau R, Vaupel JW. Ageing populations: the challenges ahead. Lancet. 2009;374(9696):1196-208.
4. Spijker J, MacInnes J. Population ageing: the timebomb that isn't? BMJ. 2013;347:f6598.
5. van Rijn RM, Robroek SJ, Brouwer S, Burdorf A. Influence of poor health on exit from paid employment: a systematic review. Occup Environ Med. 2014;71(4):295-301.
6. Carr E, Fleischmann M, Goldberg M, Kuh D, Murray ET, Stafford M, et al. Occupational and educational inequalities in exit from employment at older ages: evidence from seven prospective cohorts. Occup Environ Med. 2018.
7. Schuring M, Robroek SJ, Lingsma HF, Burdorf A. Educational differences in trajectories of self-rated health before, during, and after entering or leaving paid employment in the European workforce. Scand J Work Environ Health. 2015;41(5):441-50.
8. Dalstra JAA, Kunst AE, Borrell C, Breeze E, Cambois E, Costa G, et al. Socioeconomic differences in the prevalence of common chronic diseases: an overview of eight European countries. International Journal of Epidemiology. 2005;34(2):316-26.
9. Bartley M, Owen C. Relation between socioeconomic status, employment, and health during economic change, 1973-93. Brit Med J. 1996;313(7055):445-9.
10. Lindholm C, Burstrom B, Diderichsen F. Class differences in the social consequences of illness? J Epidemiol Commun H. 2002;56(3):188-92.
11. Burstrom B, Holland P, Diderichsen F, Whitehead M. Winners and losers in flexible labor markets: The fate of women with chronic illness in contrasting policy environments - Sweden and Britain. International Journal of Health Services. 2003;33(2):199-217.
12. Burstrom B, Whitehead M, Clayton S, Fritzell S, Vannoni F, Costa G. Health inequalities between lone and couple mothers and policy under different welfare regimes - The example of Italy, Sweden and Britain. Social Science & Medicine. 2010;70(6):912-20.
13. Bambra C, Eikemo TA. Welfare state regimes, unemployment and health: a comparative study of the relationship between unemployment and self-reported health in 23 European countries. J Epidemiol Commun H. 2009;63(2):92-8.
14. Bambra C, Pope D. What are the effects of anti-discriminatory legislation on socioeconomic inequalities in the employment consequences of ill health and disability? J Epidemiol Commun H. 2007;61(5):421-6.
15. van der Wel KA, Dahl E, Thielen K. Social inequalities in "sickness": does welfare state regime type make a difference? A multilevel analysis of men and women in 26 European countries. Int J Health Serv. 2012;42(2):235-55.
16. Reeves A, Karanikolos M, Mackenbach J, Mckee M, Stuckler D. Do employment protection policies reduce the relative disadvantage in the labour market experienced by unhealthy people? A natural experiment created by the Great Recession in Europe. Social Science & Medicine. 2014;121:98-108.
17. Dahl E, Fritzell J, Lahelma E, Martikainen J, Kunst A, Mackenbach JP. Welfare state regimes and health inequalities. In: Siegrist J, Marmot M, editors. Social Inequalities in Health: New evidence and policy implications. Oxford: Oxford University Press; 2006. p. 194-217.
18. Minton JW, Pickett KE, Dorling D. Health, employment, and economic change, 1973-2009: repeated cross sectional study. BMJ. 2012;344:e2316.

For peer review only - http://bmjopen.bmj.com/site/about/guidelines.xhtml

19. Whitehead M, Burstrom B, Dahl E, Clayton S, Diderichson F, Holland P, et al. Helping chronically ill or disabled people into work: what can we learn from international comparative analyses? Final report to the Department of Health. York: Public Health Research Consortium, 2009.
20. Gesis. GML: EU-SILC: GESIS; 2017 [Available from: https://www.gesis.org/gml/european-microdata/eu-silc/.
21. Hellevik O. Linear versus logistic regression when the dependent variable is a dichotomy. Qual Quant. 2009;43(1):59-74.
22. Barros AJ, Hirakata VN. Alternatives for logistic regression in cross-sectional studies: an empirical comparison of models that directly estimate the prevalence ratio. BMC Med Res Methodol. 2003;3:21.
23. Ukoumunne OC, Forbes AB, Carlin JB, Gulliford MC. Comparison of the risk difference, risk ratio and odds ratio scales for quantifying the unadjusted intervention effect in cluster randomized trials. Stat Med. 2008;27(25):5143-55.
24. Lee J. Odds Ratio or Relative Risk for Cross-Sectional Data. International Journal of Epidemiology. 1994;23(1):201-3.
25. Genre V, Gomez-Salvador R, Lamo A. The determinants of labour force participation in the European Union. Labour Supply and Incentives to Work in Europe. 2005:195-218.
26. Eikemo TA, Hoffmann R, Kulik MC, Kulhanova I, Toch-Marquardt M, Menvielle G, et al. How can inequalities in mortality be reduced? A quantitative analysis of 6 risk factors in 21 European populations. PLoS One. 2014;9(11):e110952.
27. van der Wel KA, Dahl E, Thielen K. Social inequalities in 'sickness': European welfare states and non-employment among the chronically ill. Social Science & Medicine. 2011;73(11):1608-17.
28. OECD. How inequality compounds over the life course. Paris: OECD Publishing, 2017.
29. Maki N, Martikainen P, Eikemo T, Menvielle G, Lundberg O, Ostergren O, et al. Educational differences in disability-free life expectancy: a comparative study of long-standing activity limitation in eight European countries. Soc Sci Med. 2013;94:1-8.
30. Cambois E, Sole-Auro A, Bronnum-Hansen H, Egidi V, Jagger C, Jeune B, et al. Educational differentials in disability vary across and within welfare regimes: a comparison of 26 European countries in 2009. J Epidemiol Community Health. 2016;70(4):331-8.
31. Bambra C. Yesterday once more? Unemployment and health in the 21st century. J Epidemiol Commun H. 2010;64(3):213-5.
32. Burgard SA, Lin KY. Bad Jobs, Bad Health? How Work and Working Conditions Contribute to Health Disparities. Am Behav Sci. 2013;57(8):1105-27.
33. Leinonen T, Pietilainen O, Laaksonen M, Rahkonen O, Lahelma E, Martikainen P. Occupational social class and disability retirement among municipal employees--the contribution of health behaviors and working conditions. Scand J Work Environ Health. 2011;37(6):464-72.
34. Robroek SJ, Rongen A, Arts CH, Otten FW, Burdorf A, Schuring M. Educational Inequalities in Exit from Paid Employment among Dutch Workers: The Influence of Health, Lifestyle and Work. PLoS One. 2015;10(8):e0134867.
35. Leijten FR, de Wind A, van den Heuvel SG, Ybema JF, van der Beek AJ, Robroek SJ, et al. The influence of chronic health problems and work-related factors on loss of paid employment among older workers. J Epidemiol Community Health. 2015;69(11):1058-65.
36. Niedhammer I, Chastang JF, David S, Kelleher C. The contribution of occupational factors to social inequalities in health: findings from the national French SUMER survey. Soc Sci Med. 2008;67(11):1870-81.
37. Navarro V, Shi L. The political context of social inequalities and health. Soc Sci Med. 2001;52(3):481-91.
38. Castellano R, Punzo G, Rocca A. Female Labour Force Participation and Selection Effect: Southern vs Eastern European Countries. Stud Class Data Anal. 2015:35-43.
39. Bambra C. Health inequalities and welfare state regimes: theoretical insights on a public health 'puzzle'. J Epidemiol Community Health. 2011;65(9):740-5.

For peer review only - http://bmjopen.bmj.com/site/about/guidelines.xhtml

40. Pirani E, Salvini S. Is temporary employment damaging to health? A longitudinal study on Italian workers. Soc Sci Med. 2015;124:121-31.
41. Benach J, Puig-Barrachina V, Vives A, Tarafa G, Muntaner C. The challenge of monitoring employment-related health inequalities. J Epidemiol Community Health. 2012;66(12):1085-7.
42. Heggebo K, Dahl E. Unemployment and health selection in diverging economic conditions: Compositional changes? Evidence from 28 European countries. Int J Equity Health. 2015;14:121.
43. Leao T, Campos-Matos I, Bambra C, Russo G, Perelman J. Welfare states, the Great Recession and health: Trends in educational inequalities in self-reported health in 26 European countries. Plos One. 2018;13(2).
44. Bartley M, Sacker A, Clarke P. Employment status, employment conditions, and limiting illness: prospective evidence from the British household panel survey 1991-2001. J Epidemiol Community Health. 2004;58(6):501-6.
45. Bockerman P, Ilmakunnas P. Unemployment and self-assessed health: evidence from panel data. Health Econ. 2009;18(2):161-79.
46. Heistaro S, Jousilahti P, Lahelma E, Vartiainen E, Puska P. Self rated health and mortality: a long term prospective study in eastern Finland. J Epidemiol Community Health. 2001;55(4):227-32.
47. Ostlin P. Occupational History, Self Reported Chronic Illness, and Mortality - a Follow up of 25586 Swedish Men and Women. J Epidemiol Commun H. 1990;44(1):12-6.
48. Jurges H. True health vs response styles: exploring cross-country differences in self-reported health. Health Econ. 2007;16(2):163-78.

For peer review only

For peer review only - http://bmjopen.bmj.com/site/about/guidelines.xhtml

Table 1 | Characteristics of the Study on Income and Living Conditions (EU-SILC) in 2014 by European region – 30-59 year olds

		Nordic (DK,FI, SE, NO, IS)	Western (DE, AT, NL, BE, LU, FR)	Anglo-Saxon (IE, UK)	Southern (CY, ES, IT, EL, PT)	Eastern (EE, LT, LV, CZ, SK, PL, HU, SI)
		n= 15,711 (%)	n=41,624 (%)	n=13,995 (%)	n= 52,961 (%)	n= 55,433 (%)
Sex	Female	50	53	53	52	54
Education	High	46	36	44	26	25
	Intermediate	42	47	29	36	64
	Low	13	17	28	38	11
Age	30-39	27	27	33	28	29
	40-49	35	36	35	37	33
	50-59	38	37	33	34	38
Employment Status	Employed	84	80	74	67	76
	Unemployed	5	6	7	16	9
	Economically inactive	3	7	10	11	6
	Retired	0	2	2	3	3
	Disabled	5	4	6	2	6
	Other	2	0	1	0	0
Chronic illness	Yes	33	30	27	21	29

For peer review only

For peer review only - http://bmjopen.bmj.com/site/about/guidelines.xhtml

Table 2 | Absolute differences in paid employment between persons with and without a chronic illness by educational level in European regions in 2014

Region in Europe	Absolute difference (%) (95% CI)		
	Within low education	Within intermediate education	Within high education
**Male**			
Nordic	32.9% (28.9-36.8)	18.6% (16.4-20.9)	11.1% (8.5-13.7)
Continental	26.1% (23.6-28.5)	17.2% (15.7-18.6)	8.2% (6.4-10.0)
Anglo-Saxon	35.3% (31.8-38.7)	17.1% (13.1-21.1)	13.4% (9.9-17.0)
Southern	21.5% (19.7-23.3)	11.2% (8.9-13.5)	5.0% (2.2-7.9)
Eastern	33.5% (30.8-36.3)	25.5% (24.2-26.7)	9.8% (7.2-12.4)
**Female**			
Nordic	31.0% (26.2-35.7)	20.1% (17.3-22.9)	9.6% (7.1-12.0)
Continental	20.4% (17.7-23.0)	15.2% (13.5-16.9)	9.4% (7.3-11.6)
Anglo-Saxon	29.8% (25.8-33.8)	22.5% (18.5-26.6)	15.8% (12.2-19.4)
Southern	10.0% (8.0-11.9)	6.5% (4.0-8.9)	5.8% (2.9-8.6)
Eastern	23.7% (20.7-26.6)	20.6% (19.3-22.0)	6.4% (4.2-8.6)

For peer review only

Table 3 | Relative differences in paid employment between persons with and without a chronic illness by educational level in European regions in 2014

Region in Europe	Relative difference (PR) (95% CI)		
	Within low education	Within intermediate education	Within high education
**Male**			
Nordic	1.61 (1.46-1.77)	1.25 (1.21-1.30)	1.13 (1.10-1.17)
Continental	1.49 (1.40-1.58)	1.24 (1.21-1.27)	1.09 (1.07-1.12)
Anglo-Saxon	1.79 (1.64-1.95)	1.25 (1.17-1.33)	1.17 (1.12-1.23)
Southern	1.43 (1.38-1.49)	1.16 (1.12-1.20)	1.06 (1.03-1.09)
Eastern	1.94 (1.78-2.11)	1.42 (1.39-1.45)	1.11 (1.08-1.15)
**Female**			
Nordic	1.65 (1.48-1.84)	1.30 (1.24-1.36)	1.12 (1.09-1.15)
Continental	1.48 (1.39-1.58)	1.24 (1.20-1.27)	1.12 (1.09-1.15)
Anglo-Saxon	1.89 (1.70-2.11)	1.44 (1.33-1.57)	1.23 (1.17-1.30)
Southern	1.28 (1.21-1.34)	1.11 (1.07-1.16)	1.08 (1.04-1.12)
Eastern	1.76 (1.62-1.92)	1.37 (1.33-1.40)	1.08 (1.05-1.10)

For peer review only

Table 4 | Trends in absolute health-related inequalities in paid employment in European regions over the period 2005-2014

Region in Europe	Trend*		
	Low education	Intermediate education	High education
**Male**			
Nordic	0.009 (0.004-0.014)	0.001 (-0.002-0.003)	0.001 (-0.003-0.005)
Continental	0.003 (-0.001-0.007)	0.001 (0.000-0.003)	-0.002 (-0.005-0.000)
Anglo-Saxon	-0.003 (-0.013-0.007)	0.005 (-0.002-0.012)	0.003 (-0.002-0.007)
Southern	**-0.005 (-0.009--0.002)**	**-0.003 (-0.006-0.001)**	**-0.006 (-0.009--0.001)**
Eastern	0.002 (-0.004-0.009)	-0.001 (-0.004-0.002)	0.000 (-0.003-0.003)
**Female**			
Nordic	0.006 (-0.003-0.015)	-0.001 (-0.005-0.003)	-0.002 (-0.007-0.002)
Continental	**0.008 (0.004-0.011)**	**0.004 (0.003-0.006)**	0.002 (-0.002-0.005)
Anglo-Saxon	**0.006 (0.000-0.013)**	**0.008 (0.002-0.014)**	0.002 (-0.005-0.008)
Southern	-0.001 (-0.004-0.002)	0.001 (-0.003-0.005)	-0.003 (-0.008-0.003)
Eastern	**0.004 (0.000-0.008)**	0.002 (-0.001-0.004)	**-0.003 (-0.005-0.000)**

* Trend describes the widening (positive value) or narrowing (negative value) of the absolute difference of proportion in paid employment between participants with and without a chronic illness

For peer review only

For peer review only - http://bmjopen.bmj.com/site/about/guidelines.xhtml

Table 5 | Trends in relative health-related inequalities in paid employment in European regions over the period 2005-2014

Region in Europe	Trend*		
	Low education	Intermediate education	High education
**Male**			
Nordic	**0.026 (0.012-0.040)**	0.002 (-0.003-0.007)	0.001 (-0.004-0.006)
Continental	**0.010 (0.001-0.020)**	0.002 (-0.001-0.005)	**-0.003 (-0.007-0.000)**
Anglo-Saxon	0.014 (-0.024-0.053)	0.014 (-0.001-0.029)	0.005 (-0.002-0.012)
Southern	-0.001 (-0.007-0.004)	-0.002 (-0.007-0.002)	**-0.006 (-0.011--0.001)**
Eastern	0.013 (-0.008-0.035)	-0.004 (-0.010-0.002)	0.000 (-0.004-0.004)
**Female**			
Nordic	0.028 (-0.004-0.060)	-0.001 (-0.008-0.006)	-0.003 (-0.009-0.002)
Continental	**0.015 (0.002-0.028)**	**0.004 (0.001-0.007)**	0.001 (-0.004-0.007)
Anglo-Saxon	0.037 (-0.003-0.077)	**0.020 (0.006-0.034)**	0.003 (-0.007-0.013)
Southern	-0.007 (-0.016-0.001)	0.003 (-0.004-0.011)	-0.003 (-0.010-0.005)
Eastern	0.010 (-0.011-0.031)	0.000 (-0.007-0.006)	-0.002 (-0.005-0.002)

* Trend describes the widening (positive value) or narrowing (negative value) of the relative difference of prevalence ratios in paid employment between participants with and without a chronic illness

For peer review only

Acknowledgement: We kindly acknowledge the help of the Microdata Access team from Eurostat for provision of the EU-SILC datasets to our research entity (2014/044/NL). We kindly thank the WORKLONG Impact Group, in particular Lars Andersen, professor at the Danish National Research Centre for the Working Environment, and Manuel Flores, economist at the Directorate for Employment, Labour and Social Affairs at the Organisation for Economic Co-operation and Development, for their comments on the draft of the paper. We also thank the organisation of the European Public Health Conference 2018, where the results of this study have been presented. The submitted abstract has been published in the European Journal of Public Health, https://doi.org/10.1093/eurpub/cky213.284 .

Contributorship: JLDS prepared the data, conducted the analysis, and drafted and revised the paper. MS and KMOH participated in the analysis and commented on the paper. AB conceptualised the study, participated in the analysis and commented on the paper. He is the guarantor. All authors approved the final version.

Funding: This work was conceived with financial support from award no. 208060001 by ZonMW within the Joint Programming Initiative More Years Better Lives (WORKLONG project) framework. Additional financial support was received through EIT Health. The sponsor had no role in the study design; in the collection, analysis, and interpretation of data; in the writing of the report; and in the decision to submit the article for publication.

Competing interests: None declared.

Ethical approval: Not required.

Data sharing: Anonymised, non-identifiable participant-level survey data are freely available for academic researchers after successful application from Eurostat.

The lead author (AB) affirms that the manuscript is an honest, accurate, and transparent account of the study being reported; that no important aspects of the study have been omitted; and that any discrepancies from the study as planned (and, if relevant, registered) have been explained.

This is an Open Access article distributed in accordance with the Creative Commons Attribution Non Commercial (CC BY-NC 4.0) license, which permits others to distribute, remix, adapt, build upon this work non-commercially, and license their derivative works on different terms, provided the original work is properly cited and the use is noncommercial. See: https://creativecommons.org/licenses/by-nc/4.0/.

For peer review only

Figure 1 – Legend

Proportion in paid employment with and without chronic illness by educational level – men (above) and women (below) in 26 European countries (2005-2014)

High, no chronic disease	Intermediate, no chronic disease	Low, no chronic disease
High, chronic	Intermediate, chronic	Low, chronic

Figure 2 - Legend

Relative inequality in paid employment among persons with a chronic illness comparing low educated persons with high educated persons

For peer review only

For peer review only - http://bmjopen.bmj.com/site/about/guidelines.xhtml

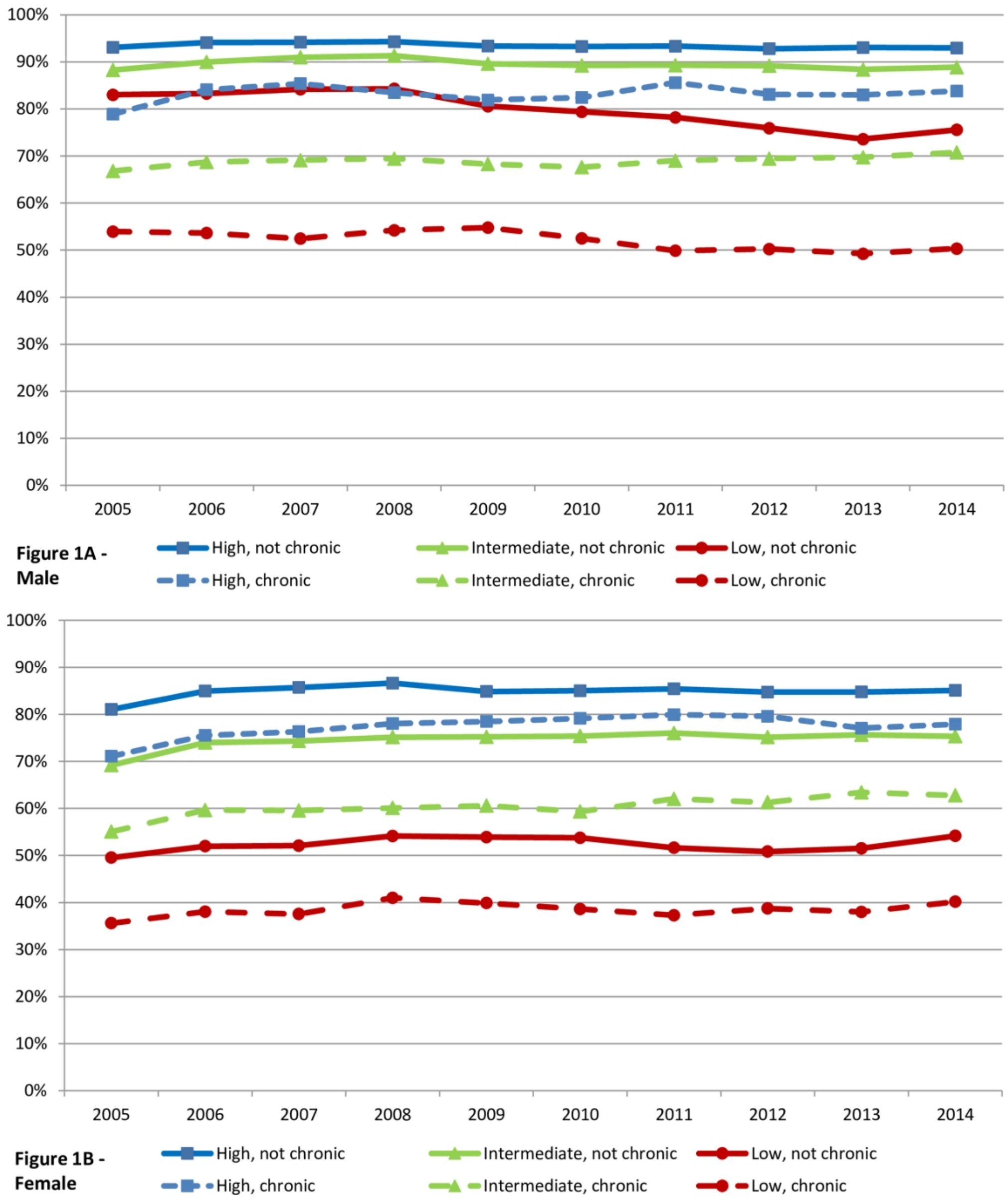

Figure 1 Proportion in paid employment with and without chronic illness by educational level – men (above) and women (below) in 26 European countries (2005-2014)

187x222mm (300 x 300 DPI)

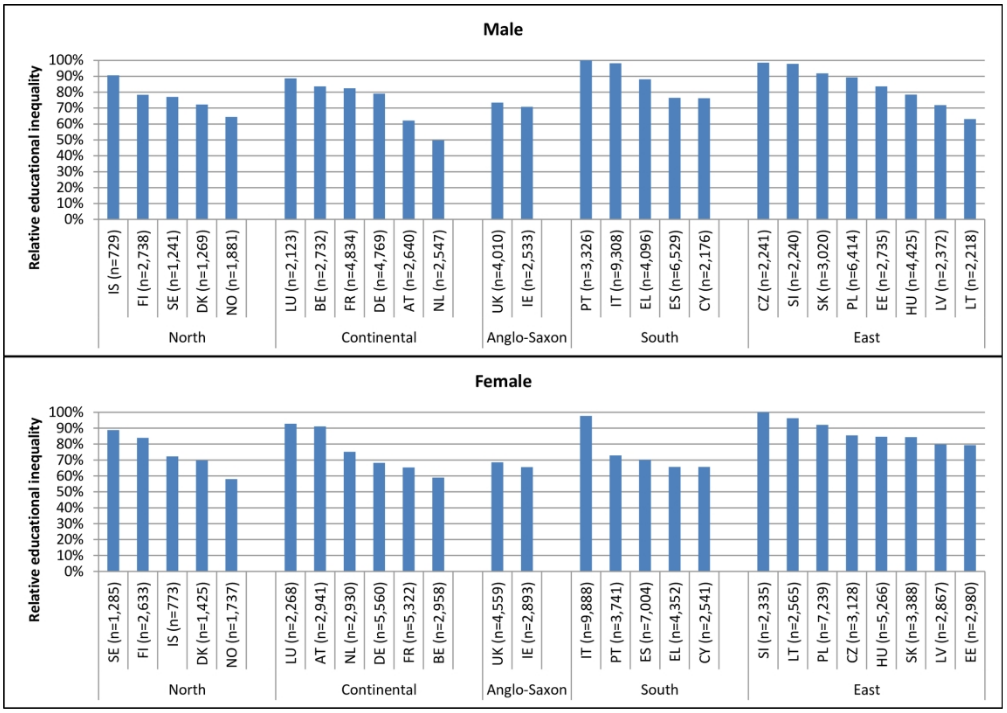

Figure 2 Relative inequality in paid employment among persons with a chronic illness comparing low educated persons with high educated persons

190x134mm (300 x 300 DPI)

Supplementary table 1 | Absolute differences in paid employment between persons with and without a chronic illness by educational level in European regions between 2005-2014 - male

Region in Europe	Absolute difference (%)									
	2005	2006	2007	2008	2009	2010	2011	2012	2013	2014
**Low education**										
Nordic	26.1	27.0	25.2	25.7	26.7	32.9	29.6	32.6	31.4	32.9
Continental	22.9	23.0	24.5	25.9	24.3	24.6	29.2	24.7	25.2	26.1
Anglo-Saxon	38.7	35.6	37.2	40.4	27.3	32.2	38.9	34.6	34.7	35.3
Southern	23.7	24.5	25.6	26.1	23.5	22.1	22.1	21.7	19.6	21.5
Eastern	28.6	31.4	36.9	35.8	30.9	32.5	32.7	35.5	32.8	33.5
**Intermediate education**										
Nordic	19.2	18.5	18.4	16.8	18.8	20.8	19.0	18.9	19.1	18.6
Continental	16.4	15.9	15.5	16.4	18.2	17.2	16.6	17.3	17.0	17.2
Anglo-Saxon	17.9	17.7	18.5	18.5	18.0	23.9	20.3	23.7	25.7	17.1
Southern	12.4	12.4	12.7	13.3	14.9	13.7	11.9	11.2	9.6	11.2
Eastern	26.0	26.6	27.5	28.7	27.1	27.8	26.9	27.5	25.3	25.5
**High education**										
Nordic	10.7	8.2	8.6	8.0	9.3	7.3	6.6	9.3	10.7	11.1
Continental	11.4	9.5	8.8	8.3	7.7	8.3	7.8	7.7	8.7	8.2
Anglo-Saxon	12.1	11.5	10.6	14.6	14.4	16.4	11.9	15.8	13.4	13.4
Southern	9.3	10.5	6.6	8.9	10.9	7.8	6.1	6.9	5.5	5.0
Eastern	10.5	11.9	9.8	12.3	11.2	12.7	10.2	12.1	11.6	9.8

For peer review only

Supplementary table 2 | Absolute differences in paid employment between persons with and without a chronic illness by educational level in European regions between 2005-2014 - female

Region in Europe	Absolute difference (%)									
	2005	2006	2007	2008	2009	2010	2011	2012	2013	2014
**Low education**										
Nordic	31.6	34.1	33.3	31.0	32.9	34.0	34.9	42.0	41.1	31.0
Continental	15.9	15.3	13.6	14.7	16.3	19.0	18.4	19.0	21.9	20.4
Anglo-Saxon	25.7	20.5	27.9	25.6	23.3	23.6	25.7	29.3	28.9	29.8
Southern	8.6	10.1	10.7	11.1	9.1	9.6	10.8	8.0	7.7	10.0
Eastern	20.2	20.7	21.9	19.4	22.1	20.5	19.8	24.6	23.4	23.7
**Intermediate education**										
Nordic	18.9	18.2	21.8	20.7	18.9	17.4	19.4	20.3	16.7	20.1
Continental	10.7	11.3	12.0	12.7	13.3	13.9	12.7	14.0	13.7	15.2
Anglo-Saxon	14.5	13.9	14.2	18.0	17.4	14.7	13.0	18.9	21.8	22.5
Southern	5.2	4.8	9.1	5.7	5.5	6.8	5.2	5.6	8.8	6.5
Eastern	19.1	19.9	21.8	21.8	22.0	22.1	21.8	22.9	21.1	20.6
**High education**										
Nordic	8.1	11.8	11.4	9.1	9.6	9.0	6.9	6.2	9.2	9.6
Continental	8.2	8.7	7.9	9.0	6.2	7.4	8.8	7.2	11.7	9.4
Anglo-Saxon	13.7	11.5	13.4	11.9	9.3	9.0	8.1	13.0	14.4	15.8
Southern	4.5	9.4	6.2	2.5	2.8	3.1	3.6	2.6	3.5	5.8
Eastern	10.0	8.2	9.9	8.6	8.0	9.2	9.1	8.4	7.5	6.4

For peer review only

Supplementary table 3 | Relative differences in paid employment between persons with and without a chronic illness by educational level in European regions between 2005-2014 - male

Region in Europe	Relative difference (PR)									
	2005	2006	2007	2008	2009	2010	2011	2012	2013	2014
**Low education**										
Nordic	1.43	1.43	1.39	1.39	1.45	1.62	1.53	1.60	1.58	1.61
Continental	1.40	1.40	1.43	1.46	1.43	1.45	1.56	1.45	1.46	1.49
Anglo-Saxon	1.86	1.74	1.83	2.05	1.66	1.88	2.14	1.95	2.02	1.79
Southern	1.40	1.42	1.43	1.44	1.41	1.38	1.40	1.41	1.38	1.43
Eastern	1.80	1.89	2.05	1.93	1.85	1.96	1.99	2.11	1.95	1.94
**Intermediate education**										
Nordic	1.26	1.25	1.24	1.22	1.26	1.29	1.26	1.26	1.26	1.25
Continental	1.23	1.22	1.21	1.22	1.25	1.24	1.23	1.24	1.23	1.24
Anglo-Saxon	1.24	1.23	1.25	1.25	1.26	1.39	1.31	1.37	1.42	1.25
Southern	1.16	1.16	1.16	1.17	1.20	1.18	1.16	1.15	1.13	1.16
Eastern	1.46	1.46	1.46	1.48	1.47	1.50	1.47	1.48	1.42	1.42
**High education**										
Nordic	1.13	1.09	1.10	1.09	1.11	1.08	1.07	1.11	1.13	1.13
Continental	1.14	1.11	1.10	1.09	1.09	1.10	1.09	1.09	1.10	1.09
Anglo-Saxon	1.15	1.14	1.13	1.18	1.19	1.22	1.15	1.21	1.17	1.17
Southern	1.11	1.13	1.08	1.11	1.13	1.09	1.07	1.08	1.07	1.06
Eastern	1.12	1.14	1.11	1.15	1.13	1.16	1.12	1.15	1.14	1.11

For peer review only

Supplementary table 4 | Relative differences in paid employment between persons with and without a chronic illness by educational level in European regions between 2005-2014 - female

Region in Europe	Relative difference (PR)									
	2005	2006	2007	2008	2009	2010	2011	2012	2013	2014
**Low education**										
Nordic	1.63	1.67	1.64	1.58	1.66	1.72	1.74	2.02	1.96	1.65
Continental	1.44	1.38	1.32	1.33	1.37	1.45	1.44	1.45	1.53	1.48
Anglo-Saxon	1.84	1.59	2.02	1.86	1.79	1.86	2.04	2.16	2.22	1.89
Southern	1.27	1.31	1.32	1.31	1.25	1.26	1.29	1.21	1.21	1.28
Eastern	1.70	1.77	1.78	1.63	1.77	1.71	1.68	1.94	1.80	1.76
**Intermediate education**										
Nordic	1.28	1.27	1.32	1.30	1.27	1.25	1.28	1.30	1.24	1.30
Continental	1.19	1.18	1.20	1.21	1.21	1.22	1.19	1.22	1.21	1.24
Anglo-Saxon	1.25	1.23	1.23	1.30	1.30	1.26	1.22	1.34	1.41	1.44
Southern	1.08	1.08	1.16	1.09	1.09	1.11	1.09	1.10	1.16	1.11
Eastern	1.38	1.39	1.42	1.41	1.42	1.43	1.41	1.44	1.38	1.37
**High education**										
Nordic	1.10	1.15	1.14	1.11	1.12	1.11	1.08	1.07	1.11	1.12
Continental	1.11	1.12	1.10	1.12	1.08	1.09	1.11	1.09	1.15	1.12
Anglo-Saxon	1.20	1.16	1.19	1.16	1.13	1.13	1.11	1.19	1.21	1.23
Southern	1.05	1.12	1.07	1.02	1.03	1.04	1.04	1.03	1.04	1.08
Eastern	1.11	1.09	1.12	1.10	1.10	1.11	1.11	1.10	1.09	1.08

For peer review only

STROBE Statement—checklist of items that should be included in reports of observational studies

	Item No	Recommendation	Reported on Page No.
**Title and abstract**	1	(*a*) Indicate the study's design with a commonly used term in the title or the abstract	1, 3
		(*b*) Provide in the abstract an informative and balanced summary of what was done and what was found	3
**Introduction**			
Background/rationale	2	Explain the scientific background and rationale for the investigation being reported	4
Objectives	3	State specific objectives, including any prespecified hypotheses	4,5
**Methods**			
Study design	4	Present key elements of study design early in the paper	6-8
Setting	5	Describe the setting, locations, and relevant dates, including periods of recruitment, exposure, follow-up, and data collection	6
Participants	6	(*a*) *Cohort study*—Give the eligibility criteria, and the sources and methods of selection of participants. Describe methods of follow-up	n.a.
		Case-control study—Give the eligibility criteria, and the sources and methods of case ascertainment and control selection. Give the rationale for the choice of cases and controls	n.a.
		Cross-sectional study—Give the eligibility criteria, and the sources and methods of selection of participants	6
		(*b*) *Cohort study*—For matched studies, give matching criteria and number of exposed and unexposed	n.a.
		Case-control study—For matched studies, give matching criteria and the number of controls per case	n.a.
Variables	7	Clearly define all outcomes, exposures, predictors, potential confounders, and effect modifiers. Give diagnostic criteria, if applicable	6
Data sources/ measurement	8*	For each variable of interest, give sources of data and details of methods of assessment (measurement). Describe comparability of assessment methods if there is more than one group	6
Bias	9	Describe any efforts to address potential sources of bias	6
Study size	10	Explain how the study size was arrived at	6
Quantitative variables	11	Explain how quantitative variables were handled in the analyses. If applicable, describe which groupings were chosen and why	6
Statistical methods	12	(*a*) Describe all statistical methods, including those used to control for confounding	7-8
		(*b*) Describe any methods used to examine subgroups and interactions	7-8
		(*c*) Explain how missing data were addressed	6
		(*d*) *Cohort study*—If applicable, explain how loss to follow-up was addressed	n.a.
		Case-control study—If applicable, explain how matching of cases and controls was addressed	n.a.
		Cross-sectional study—If applicable, describe analytical methods	n.a.

For peer review only

For peer review only - http://bmjopen.bmj.com/site/about/guidelines.xhtml

taking account of sampling strategy	
(*e*) Describe any sensitivity analyses	n.a.

Continued on next page

For peer review only

Results			Reported on Page No.
Participants	13*	(a) Report numbers of individuals at each stage of study—eg numbers potentially eligible, examined for eligibility, confirmed eligible, included in the study, completing follow-up, and analysed	9, 17
		(b) Give reasons for non-participation at each stage	n.a.
		(c) Consider use of a flow diagram	n.a.
Descriptive data	14*	(a) Give characteristics of study participants (eg demographic, clinical, social) and information on exposures and potential confounders	9, 17
		(b) Indicate number of participants with missing data for each variable of interest	n.a.
		(c) *Cohort study*—Summarise follow-up time (eg, average and total amount)	n.a
Outcome data	15*	*Cohort study*—Report numbers of outcome events or summary measures over time	n.a.
		Case-control study—Report numbers in each exposure category, or summary measures of exposure	*n.a.*
		Cross-sectional study—Report numbers of outcome events or summary measures	9,17
Main results	16	(*a*) Give unadjusted estimates and, if applicable, confounder-adjusted estimates and their precision (eg, 95% confidence interval). Make clear which confounders were adjusted for and why they were included	9, 18-21
		(*b*) Report category boundaries when continuous variables were categorized	9, 18-21
		(*c*) If relevant, consider translating estimates of relative risk into absolute risk for a meaningful time period	n.a.
Other analyses	17	Report other analyses done—eg analyses of subgroups and interactions, and sensitivity analyses	9,10,25
**Discussion**			
Key results	18	Summarise key results with reference to study objectives	11-12
Limitations	19	Discuss limitations of the study, taking into account sources of potential bias or imprecision. Discuss both direction and magnitude of any potential bias	12
Interpretation	20	Give a cautious overall interpretation of results considering objectives, limitations, multiplicity of analyses, results from similar studies, and other relevant evidence	11-12
Generalisability	21	Discuss the generalisability (external validity) of the study results	12
**Other information**			
Funding	22	Give the source of funding and the role of the funders for the present study and, if applicable, for the original study on which the present article is based	22

*Give information separately for cases and controls in case-control studies and, if applicable, for exposed and unexposed groups in cohort and cross-sectional studies.

Note: An Explanation and Elaboration article discusses each checklist item and gives methodological background and published examples of transparent reporting. The STROBE checklist is best used in conjunction with this article (freely available on the Web sites of PLoS Medicine at http://www.plosmedicine.org/, Annals of Internal Medicine at http://www.annals.org/, and Epidemiology at http://www.epidem.com/). Information on the STROBE Initiative is available at www.strobe-statement.org.

For peer review only
